# Combination cancer immunotherapy targeting TNFR2 and PD-1/PD-L1 signaling reduces immunosuppressive effects in the microenvironment of pancreatic tumors

**DOI:** 10.1136/jitc-2021-003982

**Published:** 2022-03-08

**Authors:** Xiaozhen Zhang, Mengyi Lao, Jian Xu, Yi Duan, Hanshen Yang, Muchun Li, Honggang Ying, Lihong He, Kang Sun, Chengxiang Guo, Wen Chen, Haitao Jiang, Xiaoyu Zhang, Xueli Bai, Tingbo Liang

**Affiliations:** 1 Department of Hepatobiliary and Pancreatic Surgery,the First Affiliated Hospital, School of Medicine, Zhejiang University, Hangzhou, Zhejiang, China; 2 Zhejiang Provincial Key Laboratory of Pancreatic Disease, the First Affiliated Hospital, School of Medicine, Zhejiang University, Hangzhou, Zhejiang, China; 3 Zhejiang Provincial Innovation Center for the Study of Pancreatic Diseases, Zhejiang University, Hangzhou, Zhejiang, China; 4 Zhejiang Provincial Clinical Research Center for the Study of Hepatobiliary & Pancreatic Diseases, Zhejiang University, Hangzhou, China; 5 Cancer Center, Zhejiang University, Hangzhou, China

**Keywords:** B7-H1 antigen, biomarkers, tumor, gastrointestinal neoplasms, immunotherapy

## Abstract

**Backgrounds:**

In advanced pancreatic ductal adenocarcinoma (PDAC), immune therapy, including immune checkpoint inhibitors, has limited efficacy, encouraging the study of combination therapy.

**Methods:**

Tumor necrosis factor receptor 2 (TNFR2) was analyzed via immunohistochemistry, immunofluorescence, western blotting, and ELISAs. The in vitro mechanism that TNFR2 regulates programmed cell death 1 ligand 1 (PD-L1) was investigated using immunofluorescence, immunohistochemistry, flow cytometry, western blotting, and chromatin immunoprecipitation (ChIP). In vivo efficacy and mechanistic studies, using C57BL/6 mice and nude mice with KPC cell-derived subcutaneous and orthotopic tumors, employed antibodies against TNFR2 and PD-L1. Survival curves were constructed for the orthotopic model and a genetically engineered PDAC model (LSL-Kras^G12D/+^; LSL-Trp53^R172H/+^; Pdx1-Cre). Mass cytometry, immunohistochemistry, and flow cytometry analyzed local and systemic alterations in the immunophenotype.

**Results:**

TNFR2 showed high expression and is a prognostic factor in CD8+ T cell-enriched pancreatic cancer. TNFR2 promotes tumorigenesis and progression of pancreatic cancer via dual effect: suppressing cancer immunogenicity and partially accelerating tumor growth. TNFR2 positivity correlated with PD-L1, and in vitro and in vivo, it could regulate the expression of *PDL1* at the transcription level via the p65 NF-κB pathway. Combining anti-TNFR2 and PD-L1 antibodies eradicated tumors, prolonged overall survival in pancreatic cancer, and induced strong antitumor immune memory and secondary prevention by reducing the infiltration of Tregs and tumor-associated macrophages and inducing CD8+ T cell activation in the PDAC microenvironment. Finally, the antitumor immune response derived from combination therapy is mainly dependent on CD8+ T cells, partially dependent on CD4+ T cells, and independent of natural killer cells.

**Conclusions:**

Anti-TNFR2 and anti-PD-L1 combination therapy eradicated tumors by inhibiting their growth, relieving tumor immunosuppression, and generating robust memory recall.

## Background

 Pancreatic ductal adenocarcinoma (PDAC) has a 5-year overall survival rate of less than 8% (range: 2%–9%) worldwide.^1 2^ For most patients with PDAC, the current standard of treatment is based on gemcitabine regimens, including combination chemotherapies such as FOLFIRINOX and gemcitabine/nab-paclitaxel. After this treatment, some long-term survivors began to be observed; however, the improvement in the 5-year survival rate was extremely limited.^3^,^5^ Immunotherapy has significant effects in many malignant tumors, and especially, programmed cell death 1 (PD-1)–programmed cell death 1 ligand 1 (PD-L1) blockade can induce durable tumor suppressor effects. However, pancreatic cancer stands out as a cancer that does not respond to checkpoint immunotherapy.^6^,^8^ Therefore, effective and well-tolerated immunotherapy in PDAC is urgently required.

Tumor necrosis factor receptor 2 (TNFR2) is one of only two receptors for the cytokines tumor necrosis factor (TNF) and lymphotoxin-α. TNFR2 is abundantly expressed by certain cancer cells and immune cells, mainly a subset of potent regulatory T cells (Tregs).^9 10^ TNF activates TNFR2 by recruiting a complex composed of the adapter protein TNF receptor-associated factor 2 (TRAF2) and TRAF2-associated proteins, such as TRAF1, and cellular inhibitor of apoptosis protein (cIAP1/2), which leads to a significant depletion of these complexes in the cytoplasm and thus might affect other activities of these molecules in tumor cells.^11^ High TNFR2 expression in the tumor microenvironments (TMEs) is associated with poor prognosis, and serum soluble TNF is a prognostic marker.^12^,^14^ There is now clear evidence that TNFR2 plays vital roles in the regulation of tumor progression: (1) TNFR2 expressed in malignant cancer cells promotes their growth and survival^15^; (2) TNFR2 can mediate the stimulatory activity of TNF on tumor-infiltrating Tregs marked by CD4^+^FOXP3^+^ (forkhead box O3), allowing cancer cells to survive via avoiding antitumor immune responses^16 17^; (3) TNF-TNFR2 signaling is also associated with the immunosuppressive function of CD11b^+^Gr1^+^ myeloid-derived suppressor cells.^18^ However, in the PDAC microenvironment, TNFR2’s function is not clear.

Recent studies have revealed that agonism and antagonism targeting TNFR2 is an attractive candidate for cancer therapy against several tumors.^17 19 20^ The TR75-54.7 monoclonal antibody, which is produced by Bio X cell and reportedly blocks TNFR2 from accessing its ligands TNF-α and LT-α, is the most commonly used in published research.^21 22^ However, in recent years, various novel TNFR2 agonists and antagonists have been designed and constructed. For example, Tam *et al*^17^ constructed a novel mouse anti-TNFR2 antibody (Y9) that binds to the receptor outside the TNF-binding region. Compared with the traditional anti-TNFR2 antibody, the Y9 antibody does not cause spontaneous activation or proliferation of peripheral T cells. In addition, it has been reported that a new murine monoclonal anti-TNFR2 antibody (designated TY101), as an antitumor immune reagent, when combined with HMGN1 (N1, a dendritic cell-activating TLR4 agonist) and R848 (a synthetic TLR7/8 agonist) immunotherapy, synergistically inhibits murine colon cancer effects compared with any single treatment.^23^ However, the effect of agonism and antagonism targeting TNFR2 on pancreatic cancer immunotherapy remains unknown.

The main reason for the poor effect of immunotherapy toward pancreatic cancer is its acquisition of immune privilege, the so-called ‘cold’ tumor, which is driven by the immunosuppressive microenvironment, the intertumoral heterogeneity of the stroma, poor infiltrating immune effectors, and low mutation burden.^8 24^ To address some of these obstacles, recent studies, including our previous published work, have suggested therapy comprizing combinations of immune costimulation-based strategies that could markedly augment the immune treatment effect against PDAC.^25^,^27^ In theory, targeting TNFR2 might modulate immunological features and remove the immunosuppressive PDAC TME, thus increasing anti-PD-L1 therapy’s antitumor activity. The present study aimed to assess the function and mechanism of TNFR2 in PDAC and the potential of a combination of TNFR2 blockade and PD-L1 blockage to treat pancreatic cancer.

## Methods

The complete experimental protocols are described in online supplemental material. In mass cytometry (CyTOF) analysis of immune cells, PLTTech Inc (Hangzhou, China) performed the CyTOF analyses following a previously published protocol.^28^

## Results

### TNFR2 is highly expressed and is a prognostic factor of CD8+ T cell-enriched pancreatic cancer

**Figure 1:**
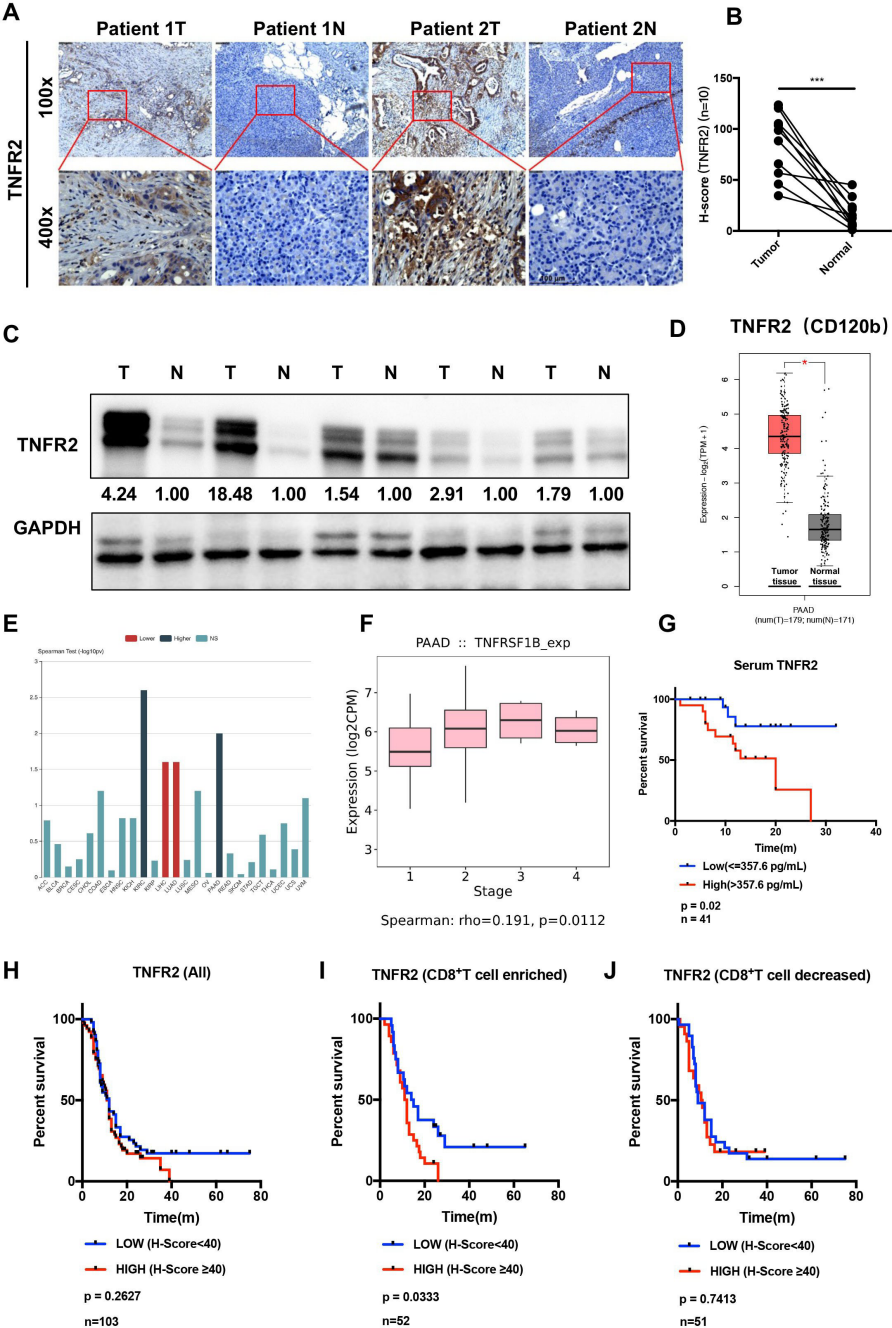
The expression and prognostic analysis of TNFR2 in pancreatic ductal adenocarcinoma (PDAC). (**A–C**) The expression profile of TNFR2 in pancreatic cancer was detected in paired tumor and normal pancreatic tissues by IHC staining (n=10) (**A and B**) and western blotting analysis (n=5) (**C**) (N, normal pancreatic tissue; T, pancreatic tumor tissue). Scale bars: 100 µm. (**D**) The relative *TNFR2* expression in pancreatic cancer and normal pancreatic tissues was analyzed using large-scale RNA-seq datasets of PDAC from the TCGA database (n=350). (**E–F**) Association between the expression of *TNFR2* and tumor stage using large-scale RNA-Seq datasets of PDAC from the TCGA database. (**G**) Overall survival (OS) of patients with pancreatic cancer with high or low concentrations of TNFR2 in their serum (n=41). (**H–J**) Overall survival (OS) of patients with all pancreatic cancer (**H**), enriched with CD8^+^ T cells (**I**) and decreased with CD8^+^ T cells (**J**), with high or low expression of TNFR2. IHC, immunohistochemistry; TCGA, The Cancer Genome Atlas; TNFR2, tumor necrosis factor receptor 2.

The expression of TNFR2 between cancerous and para-cancerous tissues from patients with PDAC was compared using immunohistochemistry (IHC). The results showed that TNFR2 expression was increased significantly in PDAC tissues compared with that in the matched para-cancerous tissues (figure 1A and B). A similar result was obtained using western blotting (WB) and immunofluorescence (IF) of TNFR2 between cancerous and para-cancerous PDAC tissues (figure 1C and online supplemental figure 1A). This conclusion was confirmed by analyses of the large-scale RNA sequencing (RNA-seq) datasets from The Cancer Genome Atlas (TCGA) database (figure 1D and online supplemental figure 1B).

**Figure 2:**
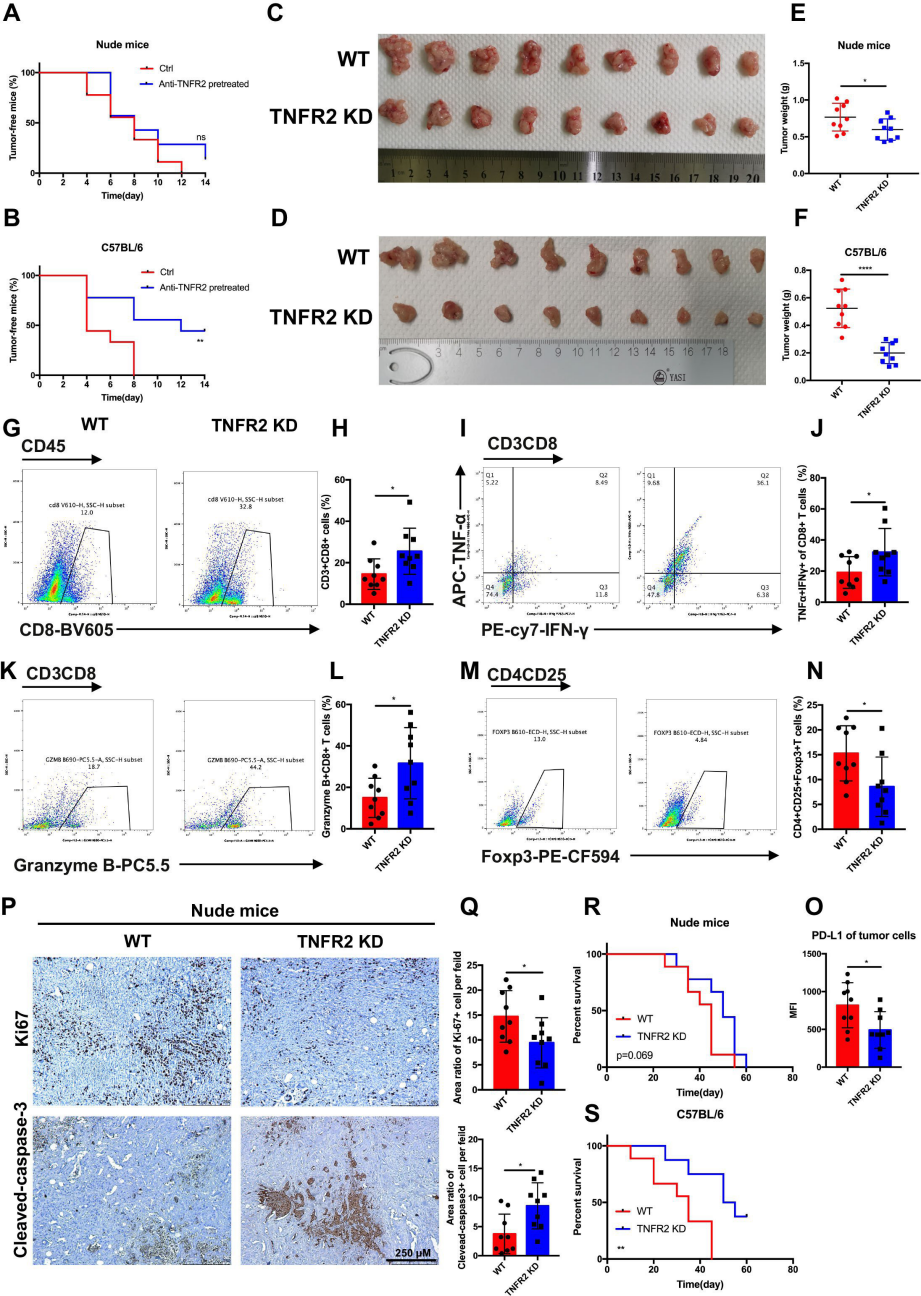
TNFR2 promotes tumorigenesis and the development of pancreatic cancer by suppressing cancer immunogenicity and partially accelerating tumor growth. (**A and B**) Pancreatic cancer cells (KPC), with or without pretreatment with anti-TNFR2 antibody (200 µg/2×10^6^ cells, 24 hours), were inoculated subcutaneously and separately into the immunocompetent and immunodeficient mice (n=7). Tumor incidence was recorded at the indicated times. (**C–F**) The visual maps of tumors and tumor weight of the immunocompetent and immunodeficient mice are shown; n=9 mice per group. (**G–N**) Representative images and statistical results of tumor-infiltrating lymphocytes (CD8+ T cells, granzyme B+CD8+ T cells, TNF-α+IFN-γ+CD8+T cells and Tregs) are shown as indicated by flow cytometry. (**O**) Flow cytometry was used to evaluate the percentage of PD-L1+ tumor cells in tumor tissues. Results are presented as mean±SD from one representative experiment. *P<0.05, **p<0.01, ***p<0.001 according to a two-tailed t-test. (**P–Q**) Representative images and statistical of the results of IHC staining of Ki-67 and cleaved-caspase-3 of *Tnfr2* knockdown tumors in immunodeficient nude mice. (**R–S**) Survival of immunocompetent and immunodeficient mice bearing TNFR2-depleted pancreatic cancer cells; n=9 mice per group. The statistical significance between wildtype (WT) and knockdown (TNFR2 KD) immunocompetent (**R**) and immunodeficient (**S**) mice was assessed using Kaplan-Meier survival curves with the log-rank test. IHC, immunohistochemistry; ns, not significant; TNFR2, tumor necrosis factor receptor 2; Tregs, regulatory T cells.

Next, analysis of a tissue microarray from patients’ tumors revealed that TNFR2 expression was frequently and positively associated with TNM stage in PDAC (p<0.0001) (left column, online supplemental table 1). In addition, analysis of TCGA data further confirmed that patients with PDAC with high expression of TNFR2 have a higher TNM stage (stage I/II and stage III/IV) (p=0.0112) (figure 1E,F). Analysis of the concentration of TNFR2 in the serum of patients with PDAC similarly demonstrated that the TNFR2 level corelated positively with the TNM stage (right column, online supplemental table 1). Furthermore, we observed that a high concentration of TNFR2 in patients’ serum was associated positively with poor prognosis in PDAC (figure 1G) (p=0.02). However, TNFR2 had no significant effect on prognosis in PDAC in the tissue microarray analysis (figure 1H), which was supported by TCGA database analysis (online supplemental figure 2A). Then, we further identified the CD8+ T cell-enriched and CD8+ T cell-decreased PDAC tissues in the microarray. Unexpectedly, TNFR2 expression correlated significantly with patient survival for tumors that expressed higher levels of CD8+ T cells, but this correlation was lost in patients whose tumors did not contain CD8+ T cells (figure 1I,J). This result was confirmed by analysis in the TCGA databases (onlinesupplemental figure 2BC). Collectively, these results indicated that not only does TNFR2 play a dominant role in progression of pancreatic cancer but also might influence the effect of immunotherapy in pancreatic cancer.

### TNFR2 promotes tumorigenesis and progression of pancreatic cancer mainly by suppressing cancer immunogenicity and partially accelerating tumor growth

To investigate the effects of TNFR2 on the tumorigenesis of pancreatic cancer in vivo, KPC cells, with or without anti-TNFR2 antibody pretreatment, were subcutaneously inoculated into immunocompetent C57BL/6 and immunodeficient nude mice, separately. We observed a marked difference in tumor incidence in the immunocompetent mice compared with that in the control group, in which anti-TNFR2 antibody pretreatment resulted in a lower incidence and longer tumor occurrence time; however, this difference was not observed in nude mice (figure 2A,B).

**Figure 3:**
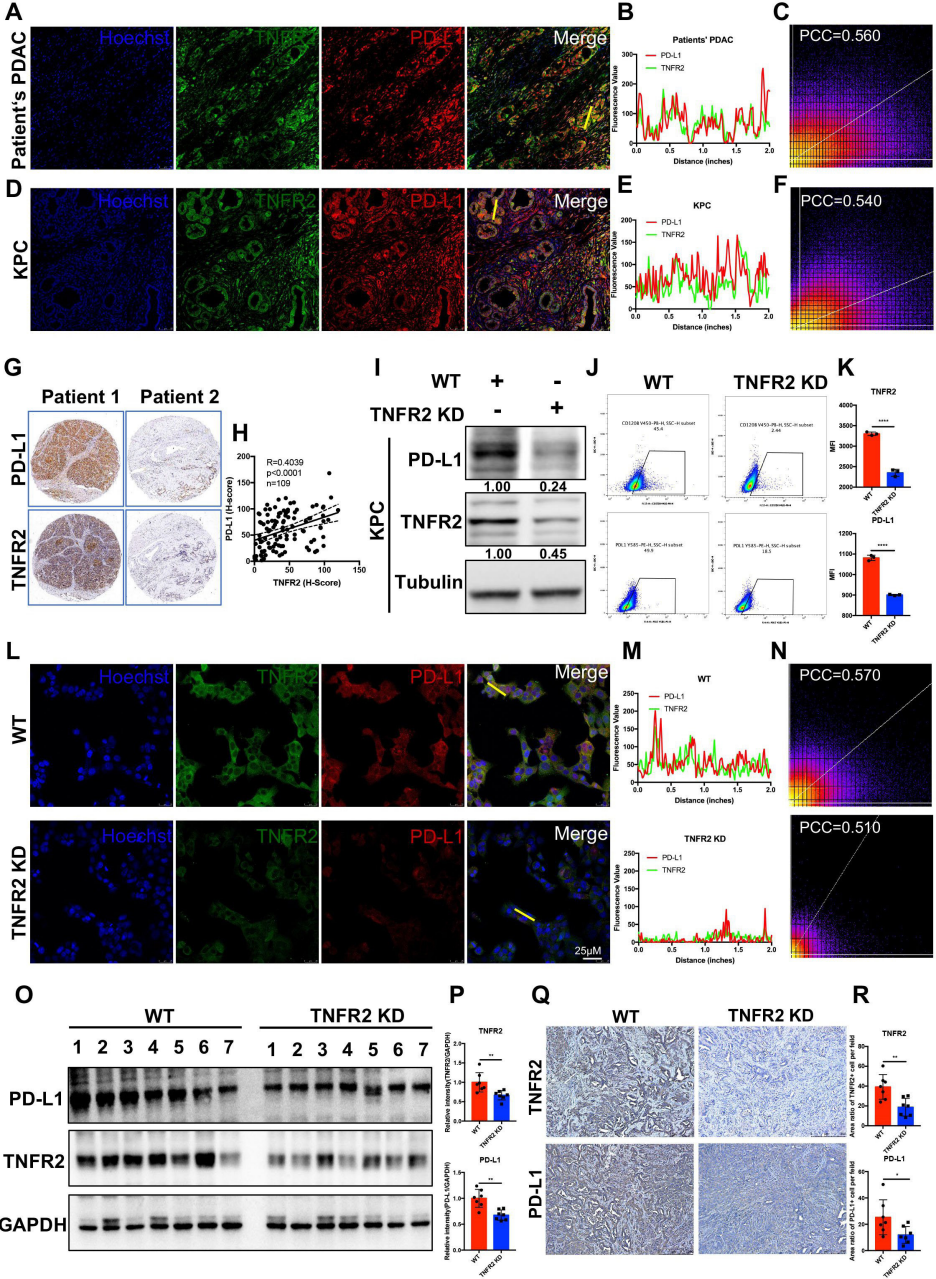
TNFR2 correlated positively with PD-L1 in pancreatic cancer. (**A–F**) Representative immunofluorescence (IF) images and the statistical results of TNFR2 and PD-L1 in tissues from patients with PDAC (**A–C**) and GEMM-KPC (LSL-Kras^G12D/+^; LSL-Trp53^R172H/+^; Pdx1-Cre) tissues (**D–F**). All images are presented at 100× magnification. (**G–H**) Representative images and the statistical results of IHC (n=109) staining of TNFR2 and PD-L1 in a tissue microarray. (**I–K**) Western blotting analysis, flow cytometry, and IF staining of PD-L1 expression in pancreatic cancer cell lines after *Tnfr2* knockdown. (**L–N**) Representative IF images and the statistical results of PD-L1 expression in pancreatic cancer cell lines after *Tnfr2* knockdown. All images are presented at 200× magnification. (**O–P**) Western blotting and the statistical results of TNFR2 and PD-L1 expression in *Tnfr2*-KD xenograft tumor samples. (**Q–R**) Representative images and the statistical results of IHC staining of TNFR2 and PD-L1 in *Tnfr2*-KD xenograft tumor samples. All images are presented at 100× magnification. *P<0.05, **p<0.01, ***p<0.001 according to a two-tailed t-test. ns, not significant; PCC, Pearson’s correlation coefficient. IHC, immunohistochemistry; PD-L1, programmed cell death 1 ligand 1; PDAC, pancreatic ductal adenocarcinoma; TNFR2, tumor necrosis factor receptor 2.

**Figure 4:**
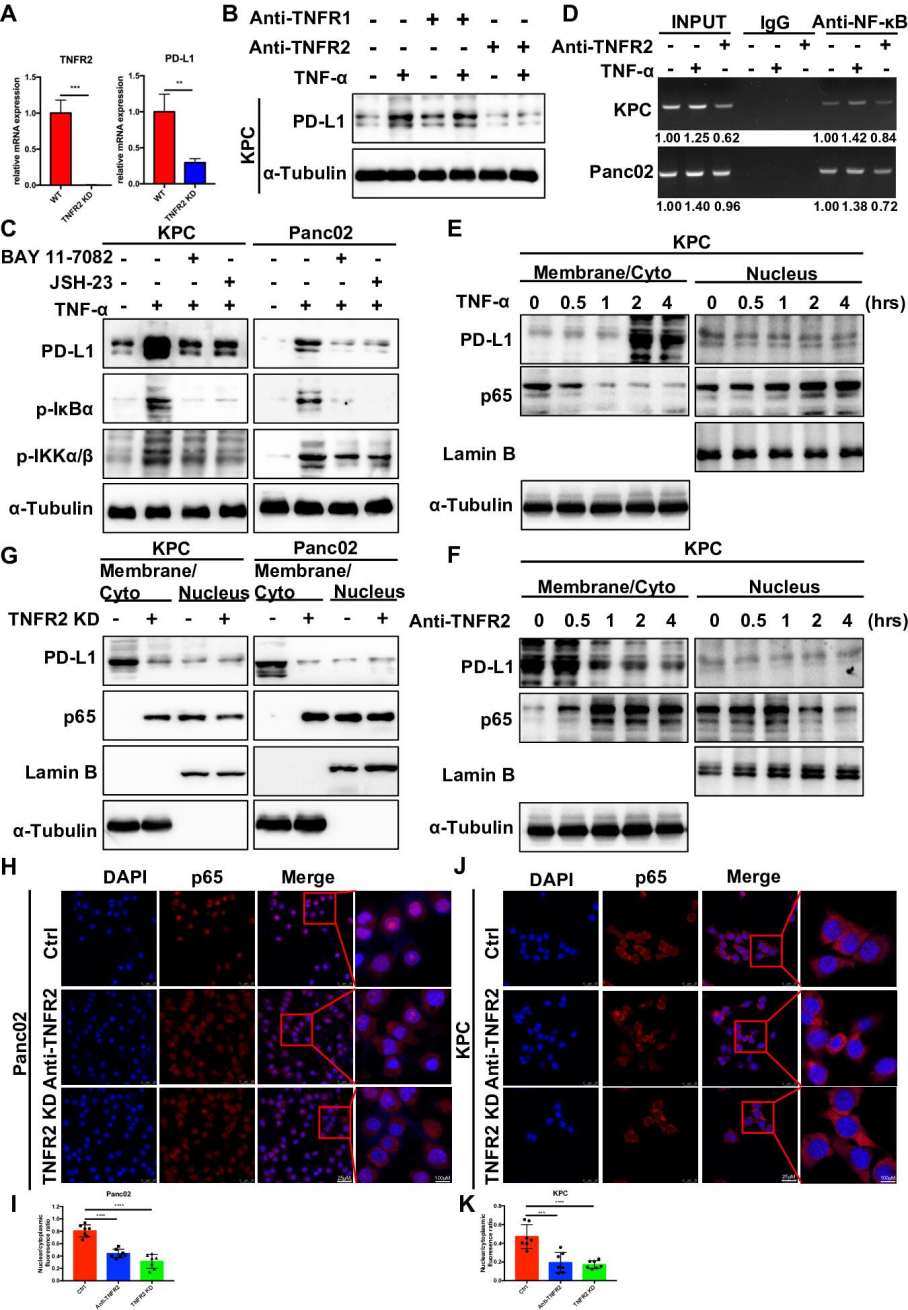
TNFR2 regulates PD-L1 expression via NF-κB p65/PD-L1. (**A**) qRT-PCR examination of the expression of PDL1 in pancreatic cancer cell with TNFR2 KD. (**B**) Western blotting analysis of PD-L1 expression under anti-TNFR1 or anti-TNFR2 blocking antibodies with and without TNF-α treatment. (**C**) Exogenous PD-L1 expression determined by western blotting analysis in KPC and Panc02 cells pretreated with NF-κB p65 inhibitors for 2 hours, followed by treatment with TNF-α for 12 hours. (**D**) Chromatin immunoprecipitation (ChIP) assay analysis of NF-κB bound potential binding site in the *CD274* (PD-L1) promotor after TNF-α treatment in PDAC cells. (**E–F**) Nuclear translocation of p65 analyzed at the indicated time points using cell fractionation in KPC cells treated with TNF-α and anti-TNFR2 antibody. (**G**) Nuclear translocation of p65 analyzed at the indicated time points using cell fractionation in KPC-*tnfr2* KD and Panc02-*tnfr2* KD cells. (**H–K**) Representative immunofluorescence (IF) images and the statistical results of nuclear translocation of p65 in TNFR2 KD and anti-TNFR2-treated pancreatic cancer cell. Scale bars: 25 µm at 100× magnification; 100 µm at 400× magnification. *P<0.05, **p<0.01, ***p<0.001 according to a two-tailed t-test. ns, not significant; PD-L1, programmed cell death 1; PDAC, pancreatic ductal adenocarcinoma; TNFR2, tumor necrosis factor receptor 2.

**Figure 5:**
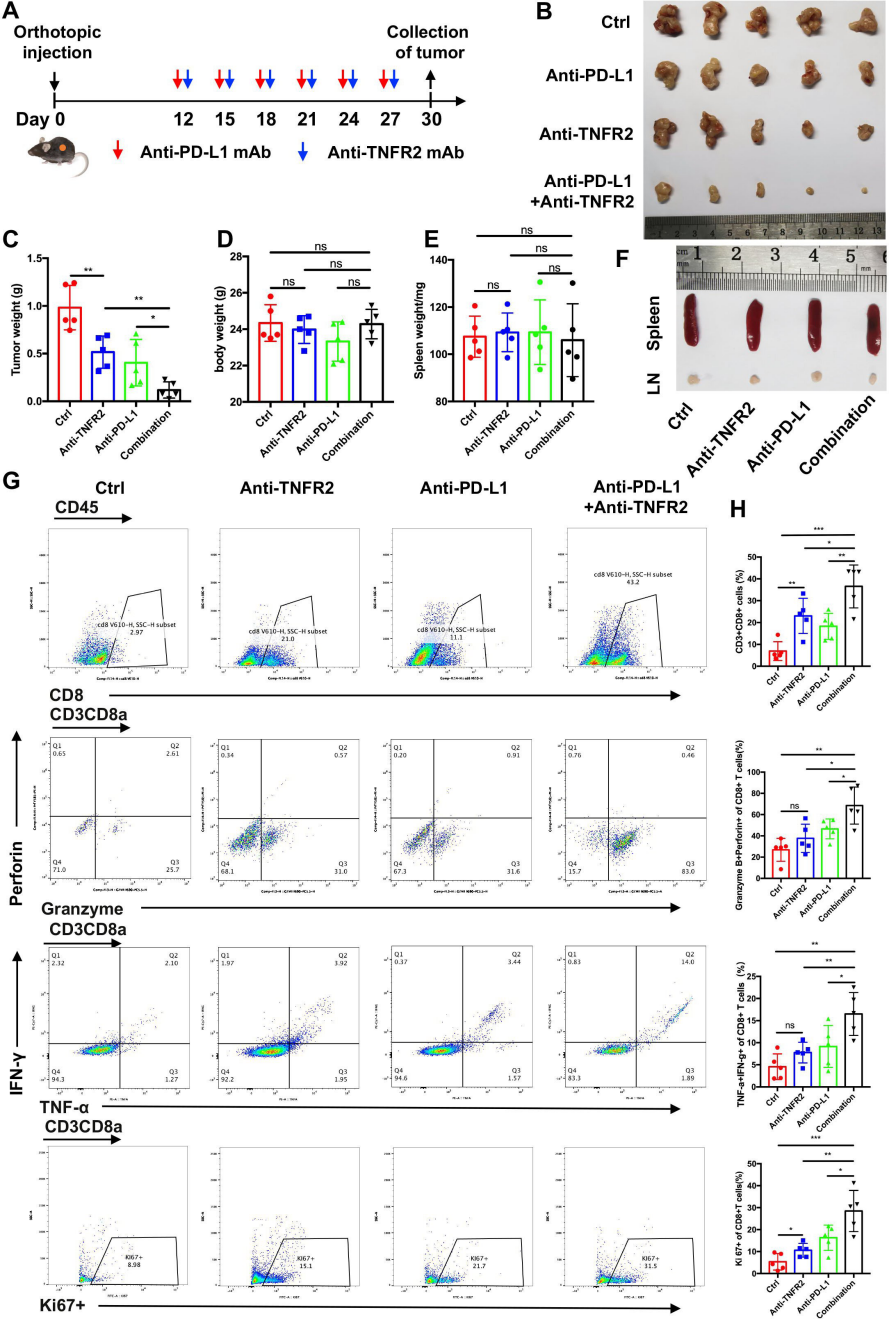
The anti-TNFR2 antibody and PD-L1 antagonist combination eradicates PDAC in orthotopic tumor-bearing mice. (**A**) Schedule of the anti-TNFR2 and anti-PD-L1 antibody combination therapy in the orthotopic model. (**B**) Representative images showing tumors harvested from mice bearing KPC cells treated with the anti-PD-L1 antibody, the anti-TNFR2 antibody, or their combination (n=5). (**C and D**) The statistical results of tumor weight and mouse body weight. (**E and F**) The statistical results of spleen weight and representative images after final treatments. (**G–H**) Flow cytometry analysis and statistical results for tumor-infiltrating lymphocytes. Data are displayed as the mean±SD of one representative experiment. *P<0.05, **p<0.01, ***p<0.001 according to a two-tailed t-test. ns, not significant; PD-L1, programmed cell death 1 ligand 1; PDAC, pancreatic ductal adenocarcinoma; TNFR2, tumor necrosis factor receptor 2.

**Figure 6:**
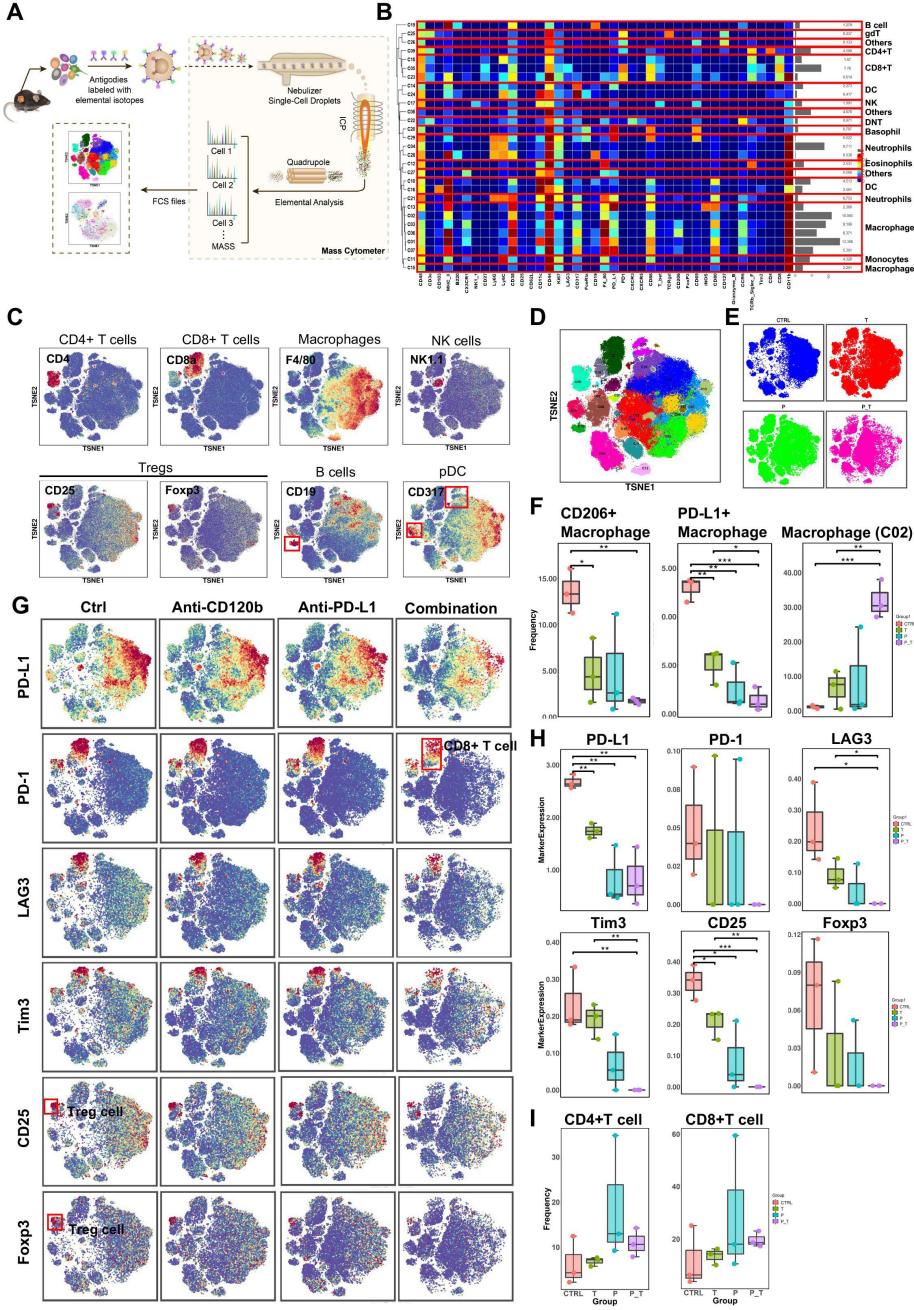
CyTOF analysis of tumor-infiltrating lymphocytes after anti-TNFR2 or anti-PD-L1 antibody therapy. (**A**) A scheme showing the experiments and CyTOF analysis of anti-TNFR2 or anti-PD-L1 combination therapy. (**B**) We identified 29 clusters, as shown in a tSNE plot. (**C**) tSNE plots were color coded for the expression of marker genes for the seven main immune cell types. (**D**) Forty-one immune markers were differentially expressed in the 29 cell clusters, as shown by a heatmap. According to typically expressed markers, certain clusters contained known cell types. (**E**) Plots of tSNE showing the distinct immune landscape of tumors in the different treatment groups. (**F**) Proportions of three immune cell types in the four treatment groups. (**G**) tSNE plots showing color-coded expression of marker genes for PD-L1, CD8^+^ T cells and Treg cells in the four treatment groups. The main types of immune cells are marked using red boxes. (**H**) Marker genes expression for PD-L1, CD8^+^ T cells and Treg cells in the four treatment groups. The data were derived from CyTOF analysis. (**I**) Proportions of CD4+ T cells and CD8+ T cells in the four treatment groups. Data are displayed as the mean±SD of one representative experiment. *P<0.05, **p<0.01, ***p<0.001 according to a two-tailed t-test. ns, not significant. CyTOF, cytometry by time of flight; PD-L1, programmed cell death 1 ligand 1; TNFR2, tumor necrosis factor receptor 2.

To further validate the effects of TNFR2 on the in vivo growth and progression of pancreatic cancer, immunocompetent C57BL/6 and immunodeficient nude mice were injected orthotopically with control or KPC-shTNFR2 (*Tnfr2* knockdown by short hairpin RNA (shRNA)) cells. The results revealed that knockdown of *Tnfr2* in KPC cells suppressed tumor growth compared with the control in immunocompetent C57BL/6. Although the *Tnfr2* knockdown group had a certain degree of reduction relative to the WT group in the immunodeficient nude mice, there was a more significant difference between the two groups in the immunocompetent C57BL/6 mice (figure 2C-F). Therefore, we further investigated the difference in immune cells between the WT and *Tnfr2* knockdown tumors in the immunocompetent models using flow cytometry. The results showed that the number and function of CD8+ T cells increased significantly in the *Tnfr2* knockdown tumors compared with those in the WT tumors, while the number of Tregs decreased in the *Tnfr2* knockdown tumors (figure 2G-N). IHC analysis in these tumors for CD3, CD8, and granzyme B confirmed this result (onlinesupplemental figure 3AB). In addition, there was a marked decrease in neoplastic PD-L1 expression (CD326+CD45-PD-L1+) in the *Tnfr2* knockdown tumors (figure 2O). Similar results were also confirmed in the subcutaneous mouse model (online supplemental figure 4A-O). Meanwhile, we evaluated the proliferation and apoptosis of tumors in immunodeficient mice using IHC analysis of Ki-67 and cleaved-caspase 3. The results showed that the proliferative ability of the tumors was inhibited, and the apoptotic area was markedly increased in the tumors in the immunodeficient mice (figure 2P,Q, onlinesupplemental figure 4PQ). Thus, these results indicated that TNFR2 regulates the growth of tumors directly. To confirm this conclusion, we performed a proliferation assay in vitro, and a similar result was obtained (onlinesupplemental figure 5AB). Furthermore, we detected the expression of survival and apoptosis pathways under TNFR2-associated change in pancreatic tumor. As expected, the downregulation of *TNFR2* in pancreatic cancer cells and anti-TNFR2 antibody decreased the levels of proteins in survival pathways, such as c-Myc, cyclin D and CDK2, and increased the levels of protein in apoptosis pathways, such as cleaved-caspase 3. The opposite result was observed in the TNF-α treatment group (online supplemental figure 5C). Moreover, to determine whether *Tnfr2* knockdown in cells affected the survival time of tumor-bearing mice, the time to reach the endpoint of each mouse was tracked and recorded, and a survival curve was plotted in the orthotopic model. The survival of C57BL/6 mice in the *Tnfr2* knockdown group increased significantly compared with that of the WT mice, while no significant difference was observed between the two groups of nude mice (figure 2R,S). In addition, a T cell killing assay was performed to test the effect of the TNFR2-associated change in pancreatic tumor PD-L1 level on the CTL activity. As expected, the downregulation of *TNFR2* in pancreatic cancer cells and anti-TNFR2 antibody in vitro made the tumor cells less resistant to lymphocytes by decreasing the numbers of inhibitory of T cells, accompanied by increasing the amount of secreted of IFN-γ and TNF-α in the T cell-mediated tumor cell-killing assay (online supplemental figure 6A-G). In addition, we found that tumor cells lacking TNFR2 are not more sensitive to cytokine-mediated death, such as that mediated by IFN-γ and TNF-α (online supplemental figure 6H). Although TNFR2 cannot regulate the expression of Fas in pancreatic cancer cells, an anti-FasL antibody could partially reverse the effect of the tumor cells’ decreased resistance to lymphocytes under *Tnfr2* KD or when treated with anti-TNFR2 antibody (online supplemental figure 6I-K). These results indicated that *Tnfr2* KD and anti-TNFR2 antibody can decrease inhibitory of T cells by downregulating PD-L1 expression to increase the ability to kill tumor cells by Fas/FasL pathways and releasing IFN-γ, TNF-α and granzyme B. Moreover, the downregulation of *TNFR2* in pancreatic cancer cells and anti-TNFR2 antibody directly inhibited the proliferation of tumor cells (onlinesupplemental figure 6A,B  D,E). Thus, we confirmed the function of TNFR2 in regulating pancreatic cancer immunogenicity and further confirmed that TNFR2 can regulate the growth of tumors directly.

Our results demonstrated that TNFR2 regulates the tumorigenesis, growth, and overall survival of pancreatic cancer mainly in an immune-dependent manner. Specifically, TNFR2 promotes tumorigenesis and progression of pancreatic cancer via cancer immunosuppression with increased number of Tregs and decreased numbers of CD3+CD8+T cells. Moreover, TNFR2 could also promote the proliferation of tumors in pancreatic cancer directly.

### In pancreatic cancer, TNFR2 positively regulates PD-L1

As shown in figure 2O, we found that the expression of neoplastic PD-L1 was significantly decreased in *Tnfr2* knockdown cells, suggesting that TNFR2 plays a vital role in cancer immunosuppression via immune checkpoint PD-L1. To test this hypothesis, we further explored the relationship between TNFR2 and PD-L1. Fluorescence double staining of TNFR2 (green) and PD-L1 (red) in patients’ PDAC tumors and GEMM KPC (LSL-Kras^G12D/+^; LSL-Trp53^R172H/+^; Pdx1-Cre) tumors was performed. We found that green fluorescence (TNFR2) and red fluorescence (PD-L1) overlapped in most positions in PDAC (Pearson’s correlation coefficient (PCC)=0.560) and KPC tumors (PCC=0.540) (figure 3A-F). Furthermore, analysis in the PDAC tissue microarray further confirmed the relationship between TNFR2 and PD-L1 expression in pancreatic tissue samples (figure 3G,H).

Next, we analyzed PD-L1 levels in KPC-*Tnfr2* knockdown cells using WB, flow cytometry, and IF. The results demonstrated that the PD-L1 levels in KPC-*Tnfr2* knockdown cells were reduced significantly (figure 3I-N). This result was confirmed in BxPC-3-*TNFR2* knockdown and SW1990-*TNFR2* cell lines (online supplemental figure 7A). Moreover, we analyzed the difference in PD-L1 levels in xenograft *Tnfr2* KD tumor samples, compared with WT tumor samples in vivo using IHC and WB. As expected, the PD-L1 levels in the *Tnfr2* KD tumor group were significantly reduced, which was similar to the in vitro results (figure 3O-R). Taken together, these findings suggested that TNFR2 correlates positively with PD-L1 and could regulate the expression of PD-L1 in pancreatic cancer.

### TNF-α upregulates PD-L1 expression through the TNFR2-p65 NF-κB pathway

To determine how TNFR2 regulates the expression of PD-L1, we found that the mRNA level of *Pdl1* was reduced in KPC-*Tnfr2* knockdown cells, similar to the protein results, indicating that TNFR2 regulates PD-L1 expression at the transcriptional level (figure 4A). Anti-TNFR1 or anti-TNFR2 blocking antibodies were used in vitro in KPC cells with the addition of TNF-α. The anti-TNFR1 antibodies did not affect the level of the PD-L1 protein in TNF-α-treated KPC cells, whereas the addition of anti-TNFR2 antibodies abolished the TNF-α-induced upregulation of PD-L1 expression in pancreatic cancer cells (figure 4B). These results indicated that TNFR2, but not TNFR1 mediates, TNF-α-induced upregulation of PD-L1 in pancreatic cancer cell. Combined with the previous conclusion, we can safely use TNF-α as a TNFR2 agonist for follow-up mechanism research to compensate for the lack of commercial TNFR2 agonists.

**Figure 7:**
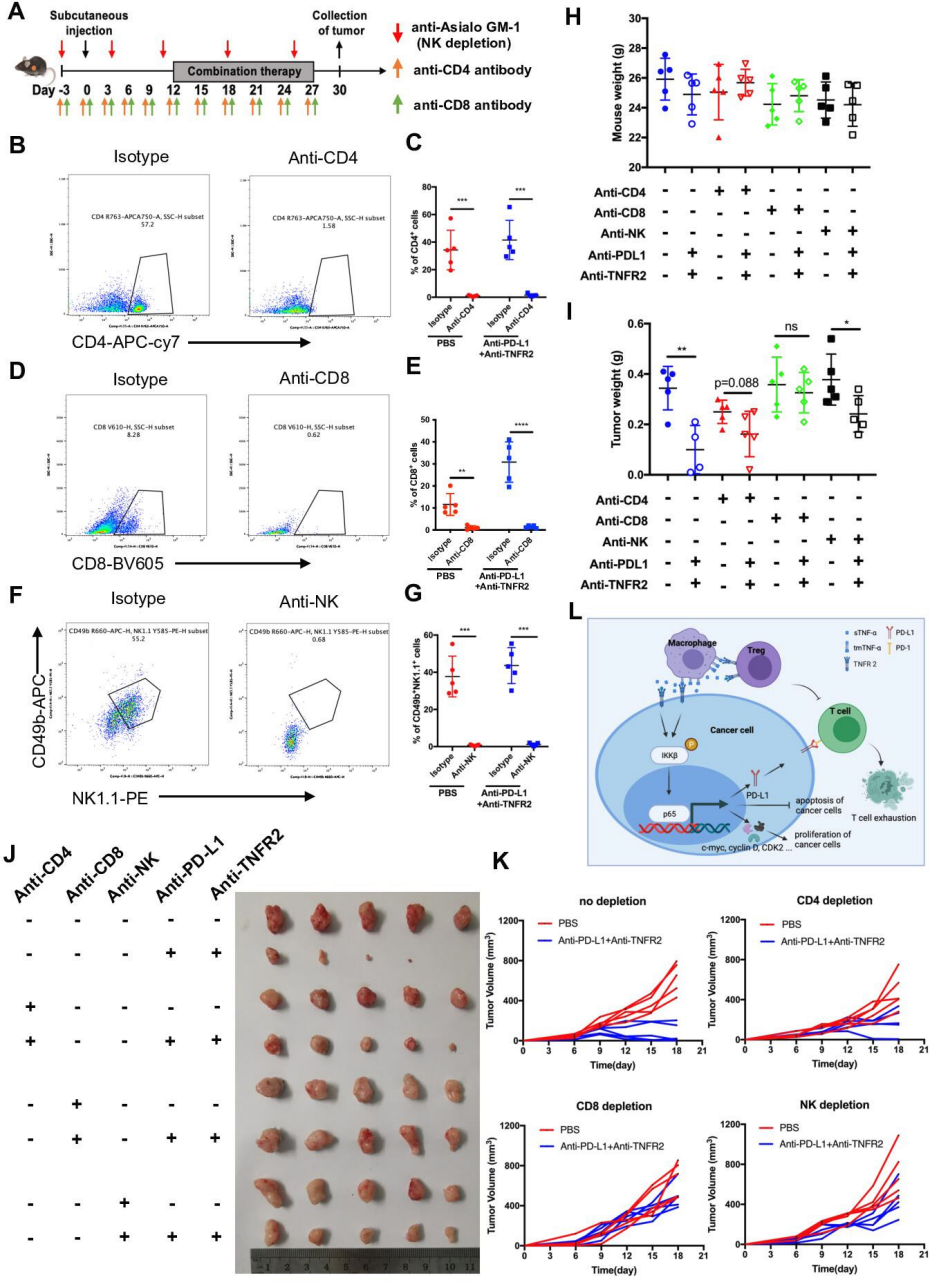
The antitumor response to combined anti-PD-L1 and anti-TNFR2 blockade depends on CD8+ T cells. (**A**) Tumor implantation and injection of antibodies schedule for immune cell (CD4^+^T cells, CD8^+^ T cells, and NK cells) depletion in mice receiving combination treatment. (**B–G**) Representative flow cytometry and quantification of NK, CD8, and CD4 staining of splenocytes to confirm immune cell depletion. (**H**) Representative images showing tumors harvested from mice bearing KPC cells treated with combination therapy after immune cell depletion (n=5). (**I–J**) The statistical results of tumor weight and mouse body weight. (**K**) Tumor growth curve of mice treated with combination therapy and antibodies for immune cell depletion. (**L**) Proposed model of the function of TNFR2 and the detailed mechanisms by which the TNFR2/NF-κB p65-PD-L1 pathway that contributes to escape from T cell immune surveillance and the TNFR2/NF-κB p65-mediated tumor growth pathway. NK, natural killer; PD-L1, programmed cell death 1 ligand 1; TNFR2, tumor necrosis factor receptor 2.

Next, to further validate the detailed mechanism of TNFR2 in PD-L1-mediated immunosuppression, we used the IκB kinase b inhibitor BAY 11–7082 and the NF-κB inhibitor JSH-23 and found that inhibitors abolished TNF-α-induced PD-L1 expression (figure 4C). In addition, NF-κB increasingly bound a potential binding site in the *CD274* promotor in TNF-α-treated PDAC cells, as determined using a chromatin immunoprecipitation assay, whereas NF-κB binding to the potential binding site in the *CD274* promoter was reduced anti-TNFR2-treated pancreatic cancer cells and in KPC/Panc02-*Tnfr2* knockdown cells (figure 4D and online supplemental figure 7B). The nuclear translocation of p65 indicated NF-κB pathway activation. Thus, we isolated nuclear and membrane or cytoplasmic fractions from KPC cells and Panc02 cells at different time points during TNF-α and anti-TNFR2 treatment. The results showed that TNF-α-induced nuclear translocation of p65 was first observed at 30 min after the start of treatment, whereas upregulation of PD-L1 began after 2 hours of treatment with TNF-α. As expected, anti-TNFR2 treatment reduced the nuclear translocation of p65 and the expression of PD-L1 (figure 4E,F, onlinesupplemental figure 7CD). Similarly, nuclear translocation of p65 and the expression of PD-L1 were reduced in KPC/Panc02-*Tnfr2* knockdown cells (figure 4G). Moreover, the result of immunofluorescent cytochemistry showed that nuclear translocation of p65 decreased in KPC/Panc02-*Tnfr2* knockdown cells and anti-TNFR2-treated pancreatic cancer cells (figure 4H-K). These data implied that TNF-α upregulates PD-L1 expression through the TNFR2-p65 NF-κB pathway.

### Combination therapy comprising anti-TNFR2 and PD-L1 antibodies eradicates tumors and increases overall survival in pancreatic cancer

As mentioned previously, TNFR2 can regulate the expression of PD-L1 in pancreatic cancer; therefore, we hypothesized that TNFR2 blockade would improve PD-L1/PD-1 blockage immunotherapy. To test this hypothesis, we first used an orthotopic model to test the efficacy of the combined therapy comprising anti-TNFR2 and anti-PD-L1 antibodies (figure 5A). As expected, the antibody combination inhibited tumor growth significantly compared with that in the control or single agent alone groups (figure 5B,C). Importantly, at the endpoint of the study, no significant changes in average mouse body weight, spleen weight, and lymph nodes size were observed (figure 5E,F), while all groups were within the normal range for liver and kidney function (online supplemental figure 8A-G). In addition, we further performed subcutaneous treatment to confirm the efficacy of the combination therapy. Similarly, the combination therapy could eliminate tumors without any evident signs of toxicity (online supplemental figure 9A-D). Moreover, we analyzed the response rate to the combination therapy through three independently repeated treatment experiments in the subcutaneous tumor model. The results showed that the response rate of the combination treatment group was significantly higher than that of the control and single-use groups (online supplemental figure 9E).

Next, we assessed the survival rate of mice treated with the antibody combination in the KPC orthotopic model. As expected, the antibody combination increased the survival of the mice significantly compared with that of the control group and treatment with either antibody alone. Notably, three mice in the combination group and one mouse in the anti-TNFR2 antibody group experienced tumor-free survival. These mice showed similar survival to healthy mice (online supplemental figure 10A). In addition, to confirm this conclusion in a genetic model, we used an autochthonous model of mutation (LSL-Kras^G12D/+^, LSL-Trp53^R172H/+^, Pdx1-Cre driven spontaneous PDAC (GEMM-KPC model)). The survival of the mice treated with the antibody combination was prolonged significantly compared with the isotype control-treated animals. However, tumor-free survival was not observed among the GEMM-KPC mice, which reinforced the implication of the function of the TME in affecting the efficacy of antibody-mediated immunotherapy (online supplemental figure 10B). These data indicated that combination therapy with anti-TNFR2 and anti-PD-L1 antibodies could increase the overall survival of patients with pancreatic cancer.

### In the microenvironment of PDAC, the combination therapy activates CD8+ T cells, reduces Treg infiltration, and induces secondary prevention and strong antitumor immune memory

To further determine how pancreas tumor progression is inhibited by combined PD-L1 and TNFR2 blockade, we conducted a series of immunophenotypic experiments. In the orthotopic model, tumors were dissected from each mouse at the end of the study, dissociated and collected, and subsequently subjected to tumor-infiltrating lymphocyte isolation and phenotypic analysis using flow cytometry. The results revealed a higher percentage of CD8+ T cells in the tumors of mice treated with the combination therapy compared with those treated with either monotherapy or those in the control group. Importantly, the combination therapy markedly increased the population of granzyme+perforin+CD8+ T cells, IFN-γ+ TNF-α+CD8+ T cells, Ki67 +CD8+ T cells, and CD4+ T cells in the tumor region (figure 5G,H, onlinesupplemental figure 11AB). Interestingly, TCF1+TIM3 CD8+T cells, which proliferated consistently in response to bead stimulus, and B cells were slightly in increased the combination group (online supplemental figure 11C-F). Moreover, the tumor-infiltrated, CD4+CD25+FOXP3+T cell population (Tregs) was also decreased significantly in the tumors of the mice treated with the combination therapy (onlinesupplemental figure 11GH). Then, IHC of immune cells were performed in subcutaneous model tumors. Similarly, the number of CD8+ T cells increased, while FOXP3+ cell numbers decreased significantly in the combination therapy-treated tumors (onlinesupplemental figure 9FG).

To gain a more comprehensive understanding of the tumor immune landscape that was affected by the treatment, tumors were dissected from treated syngeneic KPC tumor-bearing mice, and their tumor-infiltrating lymphocytes were isolated and subjected to cytometry by time of flight (CyTOF) analysis (figure 6A). Twelve samples in the orthotopic model were analyzed, and 29 clusters were identified using 41 immune markers (figure 6B). Based on typically expressed markers, certain known cell types were identified among the clusters (figure 6C,D). In general, the immune landscape of PDAC after combination therapy was altered significantly compared with that of the control group (figure 6E and online supplemental figure 12). Notably, the numbers of PD-L1 + macrophages and CD206+ (M2-polarized) macrophages (tumor-associated macrophages (TAMs)), all of which are immunosuppressive cells, were reduced significantly (figure 6F), as confirmed by flow cytometry (online supplemental figure 11I), while cluster 2 (PD-L1^neg^CD206^low^) macrophages increased remarkably in the combination group compared with those in the control and single-use groups (figure 6F). Moreover, markers of Tregs (CD25 and FOXP3) were downregulated in the combination therapy group. These results demonstrated that the combination therapy induced a decrease in the expression levels of all immune suppressive markers (PD-L1, PD-1, LAG3, and TIM-3) (figure 6G,H). However, the differences in the frequencies of CD4+ T cells and CD8+ T cells in the antibody-treated samples are not statistically significant because of individual differences within one each group (figure 6I). Thus, we performed T cell reclustering analysis in the combination therapy group. Interestingly, analysis of tumor-infiltrating lymphocytes in the T cell reclustering analysis revealed that the number of CD4+ T cells and CD8+ T cells were increased significantly in the combination group compared with those in the control and single-use groups (online supplemental figure 12E). In addition, relative to CD45+ cell clustering analysis, analysis of other tumor-infiltrating lymphocytes in the combination therapy group in the T cell reclustering analysis revealed similar results (online supplemental figure 12). Taken together, immune landscape analysis revealed that combined PD-L1 and TNFR2 blockade changes the immune-privileged TME of PDAC into an immunotherapy-favorable TME marked by decreased numbers of immune suppressive Tregs and TAMs, and increased numbers of CD4+ T cells and CD8+ T cells that might be involved in immunological memory.

To determine whether immunological memory is induced by combination therapy with anti-TNFR2 and anti-PD-L1 antibodies, tumor-free mice from the initial subcutaneous model produced by removal of the subcutaneous tumors were challenged by implantation of syngeneic PDAC cells from either the same cell line as the first implantation or a different cell line, using age-matched wild-type mice as controls. No additional therapy was provided to the rechallenged mice (online supplemental figure 13A-D). Palpation confirmed tumor disappearance at more than 100 days after the implantation of the first tumor. By contrast, in the age-matched control group, all the mice grew tumors. Some of the mice in the combination therapy group remained tumor free, compared with the control and age-matched control groups. Specifically, in the KPC rechallenge model, initial subcutaneous implantation of KPC cells, followed by combination immunotherapy-treated cells resulted in a higher incidence of a complete response (CR) to treatment (CR; tumors that were less than 60 mm^3^ and showed continued regression until study end) after secondary implantation with KPC cells, compared with the control and age-matched control groups (onlinesupplemental figure 13EH). Notably, rechallenge with KPC cells could not establish a tumor mass in mice previously cured of KPC (two of seven mice in the KPC model). Panc02 rechallenge in the model confirmed this result (online supplemental figure 13F). In the Panc02-KPC rechallenge model, initial and secondary implantation with a different cell line yielded similar results (online supplemental figure 13G). Thus, KPC tumor-derived T cells could produce T cell cross-reactivity to shared antigens of pancreatic tumors from Panc02 cells. Altogether, these results suggested that in multiple tumor models, combined treatment with anti-TNFR2 and anti-PD-L1 resulted in both immediate antitumor activity and long-term immune memory. Thus, T cells induced by previous combination therapy could eliminate rechallenge tumors via rapid immunological recall.

### The antitumor immune response induced by the combination therapy is mainly dependent on CD8+ T cells, partially dependent CD4+ T cells, and independent of natural killer (NK) cells

We depleted CD8+ T cells, CD4+ T cells, or NK cells before inoculation with KPC tumor cells and combined antibody treatment to determine which immune cell types are critical for the effects of the combination therapy (figure 7A). At the study endpoint, flow cytometry was used to confirm immune cell depletion using splenocytes from the mice (figure 7B-G). The efficacy of the anti-TNFR2 and anti-PD-L1 treatment was mostly abrogated by the administration of CD8-depleting antibodies in mice with KPC tumors, while the depletion of CD4 and NK partially inhibited the antitumor response to the combined treatment, suggesting that CD4+ T cells and NK cells play a more limited role than that of CD8+ T cells. These treatments and depletion antibodies caused no toxicity; that is, we observed no weight loss in any of the treated animals (figure 7). These results revealed that the antitumor immune response derived from the combination therapy is mainly dependent on CD8+ T cells, partially dependent CD4+ T cells, but independent of natural killer cells.

Collectively, our results demonstrated that TNFR2 promotes tumorigenesis and progression of pancreatic cancer via dual effect: suppressing cancer immunogenicity via the TNFR2-NFκB p65-PDL1 pathway and partially accelerating tumor growth via the TNFR2-NFκB-survial pathways (c-Myc, cyclin D and CDK2). Combining anti-TNFR2 and anti-PD-L1 antibodies eradicated tumors, prolonged overall survival in pancreatic cancer, and induced strong antitumor immune memory and secondary prevention by reducing the infiltration of Tregs and TAMs and CD8+ T cell activation in the PDAC microenvironment (figure 7L).

## Discussion

Although PD-1/PD-L1 immune checkpoint blockade leads to a CR of immunogenic tumors and has demonstrated clinical success, there are still challenges to identify a strategy to treat non-immunogenic tumors. These challenges might be associated closely with the strong immunosuppression induced by infiltrating immune cells, such as in pancreatic cancer.^29^ Therefore, the relief of tumor immunosuppression is very important for the immunotherapy of pancreatic cancer.

TNFR2 is a cell-surface receptor that regulates cell survival and proliferation; however, more recent research has concentrated on TNFR2’s role in regulating tumor immunosuppression.^16 17 19^ Correspondingly, targeting this receptor has emerged recently as a potential next-generation approach to cancer therapy. Anti-TNFR2 agonistic antibodies can enhance the activity of effector T cells, or destabilize inhibitory Tregs by blocking TNF/TNFR2 signaling.^30 31^

In the present study, the results demonstrated that the tumoricidal activity of a single immunotherapy antibody is insufficient; however, the anti-TNFR2 and anti-PD-L1 antibody combination showed unexpected curative effects, completely eliminating the disease and allowing long-term survival in multiple pancreatic tumor models.

Subsequent studies should investigate further how TNFR2 blockade enhances the efficacy of immunotherapy in pancreatic cancer. In that regard, TNFR2 blockade might have the effect of killing two birds with one stone: directly killing tumor cells and enhancing antitumor immune responses. We found that *Tnfr2* KD or the use of anti-TNFR2 antibodies could inhibit cancer cell proliferation directly, and induced the apoptosis of tumor cells in pancreatic cancer. Similar results have been demonstrated in other cancers.^20 32 33^ In terms of enhancing antitumor immune responses, we performed comprehensive tumor immune microenvironment analysis of tumor samples from mice treatment models using flow cytometry and CyTOF. The results revealed that the combination therapy increased the number of infiltrating effector cells into the tumor area and upregulated the tumor-killing function of CD8+ T cells while significantly decreasing the number of Tregs, CD206+macrophages (TAMs), PD-L1+macrophages, and the CD8+ T cell expression of immune checkpoints (eg, TIM-3, LAG3, and PD-1), which promoted immune suppressive effects in pancreatic cancer. Notedly, this was the first study to demonstrate the changes in macrophages under anti-TNFR2 antibody treatment. TNF-α, which promotes tumor growth and proliferation, is mainly secreted by macrophages.^34 35^ TNFR2 is one of two TNF-α receptors that transduce TNF biological activity.^36^ More details about TNFα-TNFR2-macrophages should be obtained in future research. Furthermore, combination therapy led to short-term antitumor activity and induced long-term immune memory in pancreatic cancer mouse models. In addition, the efficacy of the combination therapy to inhibit tumor progression mainly depended on CD8+ T cells, depended partly on CD4+ T cells, and was independent of NK cells in the mouse models. In terms of the molecular mechanisms of molecular this combination therapy, we found that TNFR2 levels correlated positively with PD-L1 levels, and TNFR2 could regulate PD-L1 expression in pancreatic cancer in vitro and in vivo. In addition, Lim’s study showed that TNF-α is a major factor that triggers cancer cell immunosuppression in response to T cell surveillance by stabilizing PD-L1 via the p65/CSN5 signaling axis.^37^ Thus, we also explored the role of the NF-κB p65 pathway in TNFR2-regulated PD-L1 in pancreatic cancer. Surprisingly, TNF-α induced the expression of PD-L1 through TNFR2 but not TNFR1, and we found that NF-κB -binding sites in the PD-L1 promoter region at which p65 binding is regulated by TNF-α-TNFR2 signaling axis. Thus, the mechanisms by which TNFR2 blockade enhances the efficacy of immunotherapy in pancreatic cancer are as follows: (1) direct inhibition of cancer cell proliferation via inhibiting NFκB p65/survial pathways (c-Myc, cyclin D and CDK2); and (2) relief of tumor immunosuppression by downregulating the expression of PD-L1 via the NF-κB p65/PD-L1 pathway and reducing the number of Tregs and TAMs to reinvigorate exhausted T cells.

Finally, the expression of TNFR2 in tumor tissues and soluble TNFR2 in serum was associated positively with poor prognosis (online supplemental table 1). Thus, TNFR2 could be used as an important prognostic indicator for patients with pancreatic cancer.

### Conclusions

In conclusion, the results of the present study revealed that anti-TNFR2 and anti-PD-L1 combination therapy boosted tumor eradication by inhibiting the growth of tumors, relieving tumor immunosuppression, and generating robust memory recall. The present study proposed an immunotherapy regimen that is effective against pancreatic cancer.

## Supplementary material

10.1136/jitc-2021-003982 online supplemental file 1

10.1136/jitc-2021-003982 online supplemental file 2

10.1136/jitc-2021-003982 online supplemental file 3

10.1136/jitc-2021-003982 online supplemental file 4

10.1136/jitc-2021-003982 online supplemental file 5

## Data Availability

All data relevant to the study are included in the article or uploaded as supplementary information.

## References

[1] Ferlay J, Soerjomataram I, Dikshit R (2015). Cancer incidence and mortality worldwide: sources, methods and major patterns in GLOBOCAN 2012. Int J Cancer.

[2] Siegel RL, Miller KD, Jemal A (2019). Cancer statistics, 2019. CA Cancer J Clin.

[3] Von Hoff DD, Ervin T, Arena FP (2013). Increased survival in pancreatic cancer with nab-paclitaxel plus gemcitabine. N Engl J Med.

[4] Conroy T, Desseigne F, Ychou M (2011). FOLFIRINOX versus gemcitabine for metastatic pancreatic cancer. N Engl J Med.

[5] Kamisawa T, Wood LD, Itoi T (2016). Pancreatic cancer. Lancet.

[6] Brahmer JR, Tykodi SS, Chow LQM (2012). Safety and activity of anti-PD-L1 antibody in patients with advanced cancer. N Engl J Med.

[7] Sharma P, Callahan MK, Bono P (2016). Nivolumab monotherapy in recurrent metastatic urothelial carcinoma (CheckMate 032): a multicentre, open-label, two-stage, multi-arm, phase 1/2 trial. Lancet Oncol.

[8] Morrison AH, Byrne KT, Vonderheide RH (2018). Immunotherapy and prevention of pancreatic cancer. Trends Cancer.

[9] Vanamee Éva S, Faustman DL (2017). TNFR2: a novel target for cancer immunotherapy. Trends Mol Med.

[10] He J, Li R, Chen Y (2019). TNFR2-expressing CD4^+^Foxp3^+^ regulatory T cells in cancer immunology and immunotherapy. Prog Mol Biol Transl Sci.

[11] Li X, Yang Y, Ashwell JD (2002). TNF-RII and c-IAP1 mediate ubiquitination and degradation of TRAF2. Nature.

[12] Tarhini AA, Lin Y, Yeku O (2014). A four-marker signature of TNF-RII, TGF-α, TIMP-1 and CRP is prognostic of worse survival in high-risk surgically resected melanoma. J Transl Med.

[13] Heemann C, Kreuz M, Stoller I (2012). Circulating levels of TNF receptor II are prognostic for patients with peripheral T-cell non-Hodgkin lymphoma. Clin Cancer Res.

[14] Babic A, Shah SM, Song M (2016). Soluble tumour necrosis factor receptor type II and survival in colorectal cancer. Br J Cancer.

[15] Ungewickell A, Bhaduri A, Rios E (2015). Genomic analysis of mycosis fungoides and Sézary syndrome identifies recurrent alterations in TNFR2. Nat Genet.

[16] Chen X, Bäumel M, Männel DN (2007). Interaction of TNF with TNF receptor type 2 promotes expansion and function of mouse CD4+CD25+ T regulatory cells. J Immunol.

[17] Tam EM, Fulton RB, Sampson JF (2019). Antibody-mediated targeting of TNFR2 activates CD8^+^ T cells in mice and promotes antitumor immunity. Sci Transl Med.

[18] Zhao X, Rong L, Zhao X (2012). TNF signaling drives myeloid-derived suppressor cell accumulation. J Clin Invest.

[19] Nie Y, He J, Shirota H (2018). Blockade of TNFR2 signaling enhances the immunotherapeutic effect of CpG ODN in a mouse model of colon cancer. Sci Signal.

[20] Torrey H, Butterworth J, Mera T (2017). Targeting TNFR2 with antagonistic antibodies inhibits proliferation of ovarian cancer cells and tumor-associated Tregs. Sci Signal.

[21] Leclerc M, Naserian S, Pilon C (2016). Control of GVHD by regulatory T cells depends on TNF produced by T cells and TNFR2 expressed by regulatory T cells. Blood.

[22] DeBerge MP, Ely KH, Wright PF (2015). Shedding of TNF receptor 2 by effector CD8⁺ T cells by ADAM17 is important for regulating TNF-α availability during influenza infection. J Leukoc Biol.

[23] Jiang M, Liu J, Yang D (2021). A TNFR2 antibody by countering immunosuppression cooperates with HMGN1 and R848 immune stimulants to inhibit murine colon cancer. Int Immunopharmacol.

[24] Clark CE, Hingorani SR, Mick R (2007). Dynamics of the immune reaction to pancreatic cancer from inception to invasion. Cancer Res.

[25] Mace TA, Shakya R, Pitarresi JR (2018). IL-6 and PD-L1 antibody blockade combination therapy reduces tumour progression in murine models of pancreatic cancer. Gut.

[26] Ma Y, Li J, Wang H (2020). Combination of PD-1 inhibitor and OX40 agonist induces tumor rejection and immune memory in mouse models of pancreatic cancer. Gastroenterology.

[27] Zhang X, Huang X, Xu J (2021). NEK2 inhibition triggers anti-pancreatic cancer immunity by targeting PD-L1. Nat Commun.

[28] Han G, Spitzer MH, Bendall SC (2018). Metal-isotope-tagged monoclonal antibodies for high-dimensional mass cytometry. Nat Protoc.

[29] Topalian SL, Drake CG, Pardoll DM (2015). Immune checkpoint blockade: a common denominator approach to cancer therapy. Cancer Cell.

[30] Williams GS, Mistry B, Guillard S (2016). Phenotypic screening reveals TNFR2 as a promising target for cancer immunotherapy. Oncotarget.

[31] Chen X, Oppenheim JJ (2017). Targeting TNFR2, an immune checkpoint stimulator and oncoprotein, is a promising treatment for cancer. Sci Signal.

[32] Torrey H, Khodadoust M, Tran L (2019). Targeted killing of TNFR2-expressing tumor cells and T_regs_ by TNFR2 antagonistic antibodies in advanced Sézary syndrome. Leukemia.

[33] Zhao T, Li H, Liu Z (2017). Tumor necrosis factor receptor 2 promotes growth of colorectal cancer via the PI3K/AKT signaling pathway. Oncol Lett.

[34] Riches DW, Chan ED, Winston BW (1996). TNF-alpha-induced regulation and signalling in macrophages. Immunobiology.

[35] Wang Y, Ding Q, Yen C-J (2012). The crosstalk of mTOR/S6K1 and hedgehog pathways. Cancer Cell.

[36] Medler J, Wajant H (2019). Tumor necrosis factor receptor-2 (TNFR2): an overview of an emerging drug target. Expert Opin Ther Targets.

[37] Lim S-O, Li C-W, Xia W (2016). Deubiquitination and stabilization of PD-L1 by CSN5. Cancer Cell.

