# Correction: Combination cancer immunotherapy targeting TNFR2 and PD-1/PD-L1 signaling reduces immunosuppressive effects in the microenvironment of pancreatic tumors

**DOI:** 10.1136/jitc-2021-003982corr1

**Published:** 2025-04-09

 Zhang X, Lao M, Xu J, *et al*. Combination cancer immunotherapy targeting TNFR2 and PD-1/PD-L1 signaling reduces immunosuppressive effects in the microenvironment of pancreatic tumors. *J Immunother Cancer* 2022;10:e003982. doi: 10.1136/jitc-2021–003982

This article has been corrected since it was first published online. Figure 4D was updated as the original image for the Panc02 panel was inadvertently mislabeled during figure assembly. In addition this figure 7E was updated as the CD4+T cell proportion graph in figure 7C was erroneously duplicated in figure 7E.

