# Supplementary material for: Combination cancer immunotherapy targeting TNFR2 and PD-1/PD-L1 signaling reduces immunosuppressive effects in the microenvironment of pancreatic tumors
Source: J Immunother Cancer. 2022 Mar 8;10(3):e003982. doi: 10.1136/jitc-2021-003982 (PMC8906048; doi:10.1136/jitc-2021-003982)
Supplement: online supplemental file 1 [file jitc-2021-003982supp001.pdf]

SUPPLEMENTAL MATERIALS AND METHODS

Cell cultures

KPC cells from the genetically engineered mouse model (GEMM-KPC) (LSL-Kras$^{G12D/+}$; LSL-Trp53$^{R172H/+}$; Pdx1-Cre) mice were used in the animal experiments, which were kindly donated by the laboratory of Prof. Raghu Kalluri (MD Anderson Cancer Center, Houston, TX, USA). KPC cells were grown in modified McCoy's 5A Medium (Thermo Fisher Scientific, Waltham, MA, USA) with 10% fetal bovine serum (FBS; Thermo Fisher Scientific) and 1% penicillin-streptomycin (Genom, Hangzhou, China). The Panc02, BxPC-3, and SW1990 cell lines were obtained from the American Type Culture Collection (Manassas, VA, USA), which were cultured in high-glucose Dulbecco's modified Eagle's medium (DMEM) (Gibco, Grand Island, NY, USA) supplemented with 10% fetal bovine serum and 1% penicillin-streptomycin. All cell lines were incubated at 37 °C in a humidified chamber containing 5% CO_2. The presence of mycoplasma contamination was routinely evaluated in all cells using PCR.

Cell transfection and generation of stable *Tnfr2* knockdown cells

KPC, PancO2, SW1990 and BxPC-3 cells were stably transfected with mouse *Tnfr2* knockdown or negative control lentivirus (Oobio, Shanghai, China). After incubation for 24 h, the medium was replaced with fresh medium and the transfected cells were selected by treatment with 10 μg/ml puromycin (InvivoGen, San Diego, CA, USA) for one week. The efficiency of transfection was determined using western blotting and RT-PCR to evaluate protein and mRNA expression after cell collection.

Immunoblotting

For immunoblotting, radioimmunoprecipitation assay (RIPA) buffer (P0013B Beyotime Biotechnology, Shanghai, China) containing phenylmethanesulfonyl fluoride (ST505 Beyotime Biotechnology) was used to lyse cells for 30 min on ice, followed by centrifugation at 12000 × *g* for 15 min. The supernatant was collected

containing the soluble proteins. The protein concentration was measured using the bicinchoninic acid (BCA) reagent (P0012 Beyotime Biotechnology). The lysates were heated at 100 °C in NuPAGE LDS Sample Buffer (4×) (Thermo Fisher Scientific, Waltham, MA, USA) for 3–5 min, separated using sodium dodecylsulfate polyacrylamide gel electrophoresis before being transferred electrophoretically to a polyvinylidene fluoride membrane (Millipore, Billerica, MA, USA). 5% skim milk in TBST was used to block the membranes, which were the overnight at 4 °C with primary antibodies. After washing with TBST, the membrane was incubated at 4 °C for 3 h with species-specific secondary antibodies. The signals from the immunoreactive proteins were detected using an EzWay DAB Western Blot Kit (KOMA BIOTECH, Seoul, Korea) and the correspondent bands were visualized using ChemiScopeTouch (Clinx Science Instruments, Shanghai, China). The internal controls comprised of glyceraldehyde-3-phosphate dehydrogenase (GAPDH) and α-tubulin. Abcam (Cambridge, MA, USA) proved the primary antibodies recognizing TNFR2 (1:1000 dilution, catalog ab109322), GAPDH (1:1000 dilution, catalog ab8245), α-tubulin (1:2000 dilution, catalog 11224-1-AP), and PD-L1 (1:1000 dilution, catalog 66248-1-Ig) antibodies were purchased from Proteintech (Rosemont, IL, USA). The intensity of immunoblotting bands was measured using Image J 1.8.0 (National Institutes of Health, Bethesda, MD, USA).

Immunohistochemical analysis (IHC)

IHC for CD3, CD8, Foxp3, Ki-67, cleaved-caspase-3, TNFR2, and PD-L1 was performed on mouse tumors harvested at the end of the experiment, stored in 10% neutral buffered formalin, and embedded in paraffin. Four μm-thick sections were cut and placed onto super frost+glass slides, baked for 60–90 min at 68 °C, and then deparaffinized. The antigen was retrieved using Sodium Citrate Antigen Retrieval Solution (Solarbio Life Science, Beijing, China) and the sections were boiled for 10 min, followed by incubation for 25-30 min at room temperature. 3% BSA was used to block the samples for 30 min at room temperature. Each tissue sample was treated with primary antibodies, as indicated, and incubated overnight at 4 °C, followed by

incubation with a biotin-conjugated secondary antibody for 50 min at room temperature. The target protein was visualized using a diaminobenzidine (DAB) Chromogen Kit (BDB2004; Biocare Medical; Pacheco, CA, USA), where the brown color characterized the targeted molecule. Slides were counterstained with diluted hematoxylin for 3–5 min. Representative images per tumor were captured using ImageScope software (Leica Biosystems, Wetzlar, Germany). Paraffin-embedded PDAC tissue array slides from 156 patient samples were obtained from the Department of Hepatobiliary and Pancreatic Surgery, the First Affiliated Hospital, Zhejiang University School of Medicine and incubated with antibodies against TNFR2 and PD-L1 (ab205921, Abcam). Immunohistochemical staining of the tissue array was performed by the Wuhan Servicebio technology (Wuhan, China). The immunohistochemical results were quantified by processing the images using 3D HISTECH quant center 2.1 software (3DHISTECH Kft, Budapest, Hungary).

Double immunofluorescence (IF)

KPC-*Tnfr2* knockdown and KPC-WT cells were cultured to about 70–80% confluency in 12-well plates, fixed at room temperature for 15 min using 4% paraformaldehyde, permeabilized for 15 min using 0.1% Triton X-100, and blocked for 30 min using 2% bovine serum albumin (BSA). After incubation with the primary anti-PD-L1 antibody (1:100 dilution, 14-5983-82, Thermo Fisher Scientific, Waltham, MA, USA and anti-TNFR2 antibody (1:100 dilution, catalog ab109322, Abcam) at 4 °C overnight, the cells were washed three times with phosphate-buffered saline (PBS), and incubated with a mixture of Alexa Fluor555-conjugated anti-mouse IgG (4408, 1:400 dilution, Cell Signaling Technology, Danvers, MA, USA) and Alexa Fluor488-conjugated anti-rabbit IgG (4412, 1:400 dilution, Cell Signaling Technology) at room temperature for 1 h. Then, 0.02 µg/mL Hoechst (Servicebio) was used to stain nuclei for 2 min. After three washes with PBS, A confocal laser scanning microscope TCS SP8 CARS (Leica) was used to image the cells. PDAC and KPC tissues sections from patients were deparaffinized and treated with 10 mM citrate buffer (pH 6.0) at 95–100 °C for 15 min. The slides were washed three times with PBS, treated for 10

min with 0.1% Triton X-100, and then blocked for 30 min using 10% bovine serum albumin (BSA) in PBS. Anti-TNFR2 antibody (1:100 dilution, catalog ab109322, Abcam) and anti-PD-L1 antibody (1:100 dilution, 14-5983-82, Thermo Fisher Scientific) were mixed and incubated with the slides overnight at 4 °C. Following washing, a mixture of two secondary antibodies were incubated with the slides at room temperature for 1 h, followed by Hoechst staining. Confocal laser scanning microscopy was used to image the cells.

Human tissues, serum, and clinical information

Human pancreatic adenocarcinoma cancer tissue and adjacent normal tissues specimens were obtained from the Department of Hepatobiliary and Pancreatic Surgery, the First Affiliated Hospital, School of Medicine, Zhejiang University. Paraffin-embedded PDAC tissue array slides comprising 156 samples from patients were created by the Wuhan Servicebio technology using PDAC tissue specimens from the First Affiliated Hospital, School of Medicine, Zhejiang University. Blood samples were collected from patients with PDAC before surgery, separated from serum, and stored at −80 °C. Baseline clinical data, including sex, age, cancer antigen levels (CA19-9, CA125), tumor size, tumor grade, and clinical tumor-node-metastasis (TNM) staging, were collected retrospectively. Regular follow-up was performed and the survival time of the patients was generally defined as the duration between surgery for curative resection and death. The protocol was approved by the Institutional Review Board at First Affiliated Hospital, School of Medicine, Zhejiang University, and written informed consent was obtained from all patients at the time of the enrollment.

Mice

Male 6–8-week-old C57BL/6 mice and nude mice were purchased from the Model Animal Research Center of Nanjing University and housed in specific pathogen-free conditions in cages of up to five animals. C57BL/6 and KPC mice were maintained under a 12-hour dark/12-hour light cycle with food and water provided *ad libitum*.

Animal Study

KPC cells (2×10^5 in 100 μL of serum-free McCoy's 5A), with or without pretreatment with anti-TNFR2 antibodies for 24 h, were separately and injected subcutaneously into the right flank of 6–8-week-old male C57BL/6 mice and nude mice (n = 9). Tumor incidence was recorded. KPC-*Tnfr2* knockdown (KD) cells and KPC-wild-type (WT) cells (1×10^6 in 100 μL of serum-free McCoy's 5A), were injected subcutaneously and separately into the right flanks of 6–8-week-old male C57BL/6 mice and nude mice (n = 7). Tumor growth was measured every other day using calipers. Three weeks after tumor implantation, the mice were sacrificed, and the tumor weight was determined. The tumors in the C57BL/6 mice were divided into three parts. One third was lysed to obtain a single cell suspension of tumor cells that were used for fluorescence activated cell sorting (FACS) analysis. One third was snap frozen in liquid nitrogen for further PCR and immunoblotting analysis. The remaining third was fixed in 10% neutral buffered formalin, paraffin embedded, and sectioned for further immunohistochemistry (IHC) pathological analysis. Tumors in the nude mice were harvested and used for PCR, immunoblotting, and pathological analysis. The tumor volume was calculated by measuring the longest diameter and the perpendicular short diameter. The formula to calculate the tumor volume was as follows: $1/2 \times \text{length} \times \text{width}^2$. As regards the survival experiments, KPC-*Tnfr2* KD cells and KPC-WT cells, both at a density of 1×10^6 in of 15 μL of medium, were mixed with 10 μL of Matrigel, and injected into the pancreas using a sterile insulin needle. The time of death of each mouse was tracked and recorded and a survival curve was plotted.

Tumor and spleen digestion and flow cytometry analysis

Tumor cells and immune cells from tumors and spleens were isolated for flow cytometry analysis. Tumor sample tissues were mechanically dissociated into small pieces using scissors and scalpels, placed in DMEM containing 2% FBS + collagenase IV (1 mg/mL) (17104019, Thermo Fisher Scientific) + DNase (10 μg/mL)

(D5025, Sigma-Aldrich, St. Louis, MO, USA) + Dispase (0.6 mg/mL) (17105041, Gibco) + $CaCl_2$ (3 mM) (21115, Sigma-Aldrich) and incubated at 37 °C under shaking at 220 rpm for 30 to 50 min. Digestion was stopped by the addition of DMEM containing 10% FBS. Dissociated tissues were filtered through 70 μm Cell Strainers (CLS431751-50EA, Sigma-Aldrich) and washed in phosphate-buffered saline (PBS). Single-cells were divided into two parts. Three quarters of the cells were resuspended in 36% Percoll solution (GE Healthcare, Chicago, IL, USA) containing 4% 10 × PBS and 60% serum-free DMEM and subjected to density gradient centrifugation to remove non-immune cells. The cells were stimulated with a Leukocyte Activation Cocktail (550583, BD biosciences, San Jose, CA, USA), and incubated at 37 °C for 4–6 h according to the manufacturer's instructions before being stained using a LIVE/DEAD Fixable Violet Dead Cell Staining Kit (ThermoFisher Scientific) on ice for 30 min in the dark. Next, the cells were washed with PBS, blocked using TruStain FcX™ (BioLegend, San Diego, CA, USA; anti-mouse CD16/32) antibody and stained for cell surface CD45, CD3, CD4, CD8, T cell immunoglobulin mucin 3 (TIM3), and CD25 expression on ice for 30 min in the dark in PBS plus 2% FBS. The cells were washed again with PBS, subjected to fixation and permeabilization using eBioscience™ FOXP3/Transcription Factor Staining Buffer Set (00-5523-00, ThermoFisher). In addition, the cells were stained for intracellular Granzyme B, Perforin, tumor necrosis factor alpha (TNF-α), interferon gamma (IFN-γ), transcription factor 1 (TCF1), and FOXP3 in permeabilization solution. For one quarter of the tumor cells, live and dead cells were stained and separated using LIVE/DEAD Fixable Violet Dead Cell Staining Kit and blocked using TruStain FcX™. Next, cells were stained for CD326 (EpCAM) to identify tumor cells and for PD-L1. Spleens were mechanically ground with a grinding rod and washed in PBS. Then, red blood cells were removed using 1× lysis buffer (555899, BD biosciences). In addition, single live and dead immune cells were stained and separated using the LIVE/DEAD Fixable Violet Dead Cell Staining Kit and blocked using TruStain FcX™. Next, cells were stained for CD45, CD3, CD4, and CD8 for CD4+ T cells and CD8+ T cells, and stained with CD49b and NK1.1 for

NK cells. All samples were analyzed using flow cytometry on a Beckman CytoFLEX LX (Becton Dickinson, Franklin Lakes, NJ, USA) and the flow cytometry data were analyzed by FlowJo software (Becton Dickinson).

Mouse model with antibody treatment

For the combination therapy *in vivo* experiments using a subcutaneous model, KPC cells (5×10^5 in 50 μL of serum-free McCoy's 5A) were injected subcutaneously into the right flank of 6 to 8-week-old male C57BL/6 mice. When the tumors reached 100–200 mm^3, treatment started. Mice bearing tumors were divided randomly into four groups using a computer-based random order generator: Untreated Control, treatment with anti-TNFR2 antibodies alone (200 μg each), treatment with anti-PD-L1 antibodies alone (200 μg each), and treatment with the antibody combination (n = 7) three times every week for 2 weeks. The mice received InVivoMAb anti-mouse PD-L1 (Bio X Cell, West Lebanon, NH, USA), InVivoMAb anti-mouse TNFR2 (TR75-54.7) (Bio X Cell), and IgG isotype control (Bio X Cell) via intraperitoneal injection. Before each administration, tumor growth was measured using calipers, and the tumor size was recorded. The tumors were measured between 08.30 am and 12.30 pm and the measuring order was randomized daily, with each animal tested at a different time each test day. For each animal, three different investigators were involved as follows: A first investigator prepared the treatment antibody with the number not labeled. A second investigator administered the treatment based on the randomization table, and a third investigator measured the tumor size using calipers. After the experiment, the tumor was harvested and divided into three parts as above. For the combination therapy *in vivo* experiments using an orthotopic model, a small left side abdominal incision near the spleen was made, and the pancreas was found and identified in front of the right side of the spleen. KPC cells at a density of 5×10^5 in 15 μL of serum-free McCoy’s 5A mixed with 10 μL of Matrigel were injected into the pancreas using a sterile insulin needle. The treatment started when the tumors reached 100–200 mm^3, as detected by *in vivo* imaging. Tumor-bearing mice were randomly divided into four groups and treated

with an intraperitoneal injection of anti-TNFR2 antibodies (100 μg each) and anti-PD-L1 (200 μg each), either alone or in combination (n = 5) three times every week for 2 weeks, plus an untreated control. After the experiment, the tumors were harvested and divided into three parts as above. The animals were excluded if the animal died prematurely. For the survival experiments of the orthotopic model, the KPC cells were injected as mentioned above. The treatment started when the tumors reached 100–200 mm^3, as detected by *in vivo* imaging. Tumor-bearing mice were randomly divided into four groups and treated with an intraperitoneal injection of anti-TNFR2 antibodies (100 μg each) and anti-PD-L1 antibodies (200 μg each), either alone or in combination (n = 7) three times every week until mice were near death and met prespecified early removal criteria approved by the IACUC. The time of death of each mouse was tracked and recorded, and a survival curve was plotted. For the survival experiments of the GEMM-KPC model, the schedule of treatment was the same as that of the orthotopic model. There were no exclusions of any animals in each experimental group.

Mass cytometry (CyTOF) analysis of immune cells

Briefly, DNAase, collagenase IV, and hyaluronidase (Sigma-Aldrich) were used to dissociate tumor tissue into single cells. Percoll density gradient media (Sigma-Aldrich) was used to enrich immune cells, and ACK Lysing Buffer (Sigma-Aldrich) was used to remove red blood cells. Samples were stained and blocked for 30 min using an in-house developed panel of mixed surface antibodies (Supplementary Table 3), followed by overnight fixation. The cells were then permeabilized and incubated with a mixture of intracellular antibodies. The cells were washed, and a CyTOF system (Helios, Fluidigm, San Francisco, CA, USA) was used to detect the signals. Immune cell types were identified using nonlinear dimensionality reduction [t-distributed stochastic neighbor embedding (tSNE)], and then subjected to density clustering.

***In vivo* antibody depletion of immune cell subsets**

CD8+ T cells were depleted using 200 μg of anti-CD8 monoclonal antibodies (mAbs) clone 53-6.7 (Bio X Cell, InVivoPlus grade) by intraperitoneal injection (i.p.) three days before subcutaneous inoculation with KPC tumor cells. CD4+ T cells were depleted using 200 μg of anti-CD4 clone GK1.5 (Bio X Cell, InVivoPlus grade) by i.p. three days before subcutaneous inoculation with KPC tumor cells. NK cells were depleted using 50 μL of polyclonal anti-Asialo GM1 (Ultra-LEAF grade, BioLegend) by tail intravenous injection three days before KPC tumor cell inoculation. Depletion antibodies were given every three days throughout the study.

T cell-mediated tumor cell killing assay

$CD8^+$ T cells were isolated from the mouse spleen using a $CD8^+$ T cell isolation kit, an LS Column, and a MidiMACS™ Separator. Isolated $CD8^+$ T cells were stained fluorescently using CD8- Phycoerythrin (PE) and CD3-fluorescein isothiocyanate (FITC) and then analyzed using flow cytometry to confirm their phenotype. The isolated CD8+ tumor infiltrating lymphocytes (TILs) were activated using Dynabeads Mouse T-Activator CD3/CD28 (catalog 11456D, Thermo Fisher Scientific) for 3 days according to the manufacturer's protocol. T cells were activated with IL-2 (10 ng/mL) in the whole experiment. Tumor cells were allowed to adhere to the plate overnight and were incubated for 48 h with activated $CD8^+$ T cells both with and without anti-TNFR2 antibody (100 μg/mL). KPC-*Tnfr2* KD cells and KPC-WT cells were incubated overnight and allowed to adhere to the culture plates, and then treated with isolated activated $CD8^+$ T cells and incubated for 48 h. The ratio between tumor cells and $CD8^+$ T cells was 1:8. T cells and cell debris were removed by PBS washing. The remaining living cancer cells were fixed with 4% paraformaldehyde, stained with 0.5% crystal violet, and quantified using a spectrophotometer at OD 570 nm.

Cell suppression assays

For CFSE suppression assays, $CD8^+$ T cells were collected from T cell-mediated tumor cell killing assay, and suppression of cell division was assessed by FACS analysis of CD8 counts versus CFSE.

Statistics

SPSS (version 20, IBM Corp., Armonk, NY, USA) and GraphPad Prism software program (GraphPad Inc., La Jolla, CA, USA; version 7.0) were used to perform the statistical analyses. Data from at least three biological replications are presented as mean ± SD. To compare the differences between two groups, we used Mann-Whitney U or two-sided Student's t-tests as appropriate. To compare the differences among three or more groups, we used one way analysis of variance (ANOVA). To analyze the correlation between variables, we used Spearman's rank correlation. The overall difference in the data at the endpoint was assessed using Student's *t*-tests to evaluate the tumor growth. The Kaplan–Meier method and the Gehan–Breslow–Wilcoxon test were used to analyze the difference in survival curves. A P value < 0.05 was considered statistically significant.

SUPPLEMENTAL FIGURE LEGENDS

Supplementary Figure 1. Expression profiles of TNFR2 among multiple cancer types.

(A) Expression profile of TNFR2 in pancreatic cancer was detected in paired tumor and normal pancreatic tissues using IF staining (N: Normal pancreatic tissue; T: Pancreatic tumor tissue). Scale bars, 75 μm. (B) The expression profiles of TNFR2 in multiple cancer types from the TCGA database.

Supplementary Figure 2. TNFR2 is a prognostic factor of immune "hot" PDAC.

(A-C) Overall survival (OS) of patients with all pancreatic cancers (A), those enriched

with $CD8^+$ T-cells (B), and those with decreased with $CD8^+$ T-cells (C) with high or low expression of *TNFR2* from the TCGA database.

Supplementary Figure 3. Analysis of markers of tumor-infiltrating lymphocytes in *Tnfr2* knockdown tumors.

(A-B) Representative images and statistical results of IHC staining of TNFR2, CD3, CD8 and Granzyme B of *Tnfr2* knockdown tumors in immunocompetent mice.

Supplementary Figure 4. TNFR2 promote tumorigenesis and development of pancreatic cancer by suppressing cancer immunogenicity in subcutaneous mice model.

(A-F) The visual maps of tumors, tumor growth curve and tumor weight of the immunocompetent and immunodeficient mice is shown; n = 7 mice per group. (G-N) Representative images and statistical results of tumor-infiltrating lymphocytes (CD8+ T cells, granzyme B+ CD8+T cells, Perforin+CD8+T cells and Tregs) are shown as indicated by flow cytometry. (O) Flow cytometry was used to evaluate the percentage of PD-L1+ tumor cells in tumor tissues. Results are presented as mean ± SD from one representative experiment. ns: not significant, $*P < 0.05$, $**P < 0.01$, $***P < 0.001$ according to a two-tailed t-test. (P-Q) Representative images and statistical results of IHC staining of Ki-67 and cleaved-caspase-3 of *Tnfr2* knockdown tumors in immunodeficient nude mice.

Supplementary Figure 5. TNFR2 directly regulates the growth of pancreatic cancer by TNFR2-NF-κB p65 pathway.

(A-B) Effects of *Tnfr2* knockdown and anti-TNFR2 antibodies on the proliferation of

KPC cells using the CCK-8 assay. (C) Western blotting analysis of the proteins expression about survival pathways independent of T cells under TNF-α treatment, anti-TNFR2 blocking antibodies and KPC-*Tnfr2* KD.

Supplementary Figure 6. TNFR2 inhibits the T cell-mediated pancreatic cancer cell-killing effect *in vitro*.

(**A-F**) Representative images and statistical result of a T cell-mediated cancer cell-killing assay. KPC cells with *TNFR2* knockdown cocultured with activated T cell for 48 h and subjected to crystal violet staining. The ratio of tumor cells to T cells was 1:8. Representative images of live cells are shown (**A**) and were further quantified (**B**). The relative suppression index of CD8+T cells, stained with CFSE were further quantified (**C**). KPC cells pretreated with the anti-TNFR2 antibody for 24 h were cocultured with activated T cells for 48 h and subjected to crystal violet staining. The ratio of tumor cells to T cells was 1:8. The representative images of live cells are shown (**D**) and were further quantified (**E**). The relative suppression index of CD8+T cells, stained with CFSE were further quantifie**d (F)**. Results are presented as mean ± SD from one representative experiment. $*P < 0.05$, $**P < 0.01$, $***P < 0.001$ by two-tailed t-test; ns: not significant. (G) qRT-PCR examination of the expression of IFN-γ and TNF-α in CD8+T cells cultured with KPC WT and KPT-*Tnfr2* KD. (H) The tumor growth assay of KPC treated with IFN-γ and TNF-α under TNFR2 KD. (I) Western blotting analysis of Fas expression under TNF-α treatment, anti-TNFR2 blocking antibodies and KPC-*Tnfr2* KD. (J-K) Statistical result of a T cell-mediated cancer cell-killing assay. KPC-*Tnfr2* KD cocultured with activated T cell pretreated with anti-FasL antibody for 48 h and subjected to crystal violet staining (J). KPC cells pretreated with the anti-TNFR2 antibody for 24 h were cocultured with activated T

cells pretreated with anti-FasL antibody for 48 h and subjected to crystal violet staining (K).

Supplementary Figure 7. TNFR2 positively regulates PD-L1 in PDAC tumor cells via NF-κB p65/PD-L1.

(A) Western blotting analysis of PD-L1 expression in pancreatic cancer cell lines after TNFR2 knockdown. (B) Chromatin immunoprecipitation (ChIP) assay analysis of NF-κB bound potential binding site in the CD274 promotor in TNFR2 KD pancreatic cancer cells. (C-D) Nuclear translocation of p65 analyzed at the indicated time points using cell fractionation in Panc02 cells treated with TNF-α and anti-TNFR2 antibody.

Supplementary Figure 8. Comparison of anti-TNFR2 or anti-PD-L1 antibodies' toxicity profiles after long-term exposure in C57/BL6 mice.

(A-G) The anti-TNFR2 antibody and anti-PD-L1 antibody combination therapy's toxicity profile was compared using liver and kidney function tests.

Data from one representative experiment are depicted as the mean ± SD. ns: not significant. *$P < 0.05$, **$P < 0.01$, ***$P < 0.001$ by a two-tailed t-test.

Supplementary Figure 9. PD-L1 antagonist combined with anti-TNFR2 antibodies eliminates PDAC in a subcutaneous model.

(A-B) Representative images and tumor growth curve of tumors harvested from mice bearing KPC cells treated with anti-TNFR2 antibody, anti-PD-L1 antibody, or their combination (n = 7). (C-D) Mouse weight and tumor body weight. (E) Response rates from three independent experiments. Response was defined as more than 90% reduction in tumor weight as compared with control tumors. (F-G) Representative

images and statistical results for CD8, Foxp3, Ki-67, and cleaved-caspase-3 in the subcutaneous model tumor with combination therapy. Data from one representative experiment are depicted as the mean ± SD. ns: not significant. *P < 0.05, **P < 0.01, ***P < 0.001 by a two-tailed t-test.

Supplementary Figure 10. Overall survival of mice bearing tumor with anti-TNFR2 or anti-PD-L1 combination therapy.

(A) Survival curve of orthotopic tumor implantation mice. KPC-orthotopic mice were treated with anti-TNFR2 antibody (100 μg/each), anti-PD-L1 antibody (200 μg/each), or their combination (n = 7) until the mice were at the point of death and met the prespecified early removal criteria approved by the IACUC. Kaplan-Meier survival curves constructed using the log-rank test for significance between the isotype control and anti-TNFR2/PD-L1 antibodies, anti-TNFR2 antibody and combination, anti-PD-L1 and combination groups, respectively.

(B) Tumor-bearing GEMM mice survival curve. When the solid tumor was palpable, the treatments were commenced. GEMM-KPC mice were treated with anti-TNFR2 antibody (100 μg /each), anti-PD-L1 antibody (200 μg/each), or their combination (n = 5) until the mice were at the point of death and met the prespecified early removal criteria approved by the IACUC. Kaplan-Meier survival curves constructed using the log-rank test for significance between the isotype control and anti-TNFR2/PD-L1 antibodies. *P < 0.05, **P < 0.01, ***P < 0.001, ns: not significant.

Supplementary Figure 11. Flow cytometry analysis of tumor-infiltrating lymphocytes of anti-TNFR2 or anti-PD-L1 combination therapy in orthotopic tumor-bearing mice.

(A-H) Representative images and statistical results of flow cytometry analysis for CD4+T cells, TCF1+TIM3-CD8+T cells, B cells and Tregs. (I) The statistical results of flow cytometry analysis for CD206+macrophages and PD-L1+macrophages. Data are displayed as the mean ± the standard deviation (SD) of one representative experiment. ns: not significant, *P < 0.05, **P < 0.01, ***P < 0.001 according to a two-tailed t-test.

Supplementary Figure 12. CyTOF analysis of tumor-infiltrating lymphocytes using antibodies recognizing TNFR2 and PD-L1 in T cell re-clustering analysis

(A) Forty one differentially expressed immune markers in 16 cell clusters from the T cell re-clustering analysis displayed using a heatmap. Using typically expressed markers, known cell types were identified as certain clusters. (B) tSNE plot of 16 identified clusters. (C-D) tSNE plots showing distinct immune landscape of tumors in different treatment groups. (E) In the four treatments group, the tSNE plots are color-coded for marker gene expression for $CD8^{+}$T cells and Treg cells. (F-G) Expression of marker genes for CD4+T cells and CD8+T cells in the four treatment groups. Data were derived from CyTOF. Data from one representative experiment are depicted as the mean ± SD. ns: not significant. *P < 0.05, **P < 0.01, ***P < 0.001 by a two-tailed t-test.

Supplementary Figure 13. Tumor rechallenge and immune memory after anti-TNFR2 and anti-PD-L1 immunotherapy

(A-C) Tumor rechallenge schedule and immune memory in the KPC and PancO2 models. (D) Timings of the implantation of de novo PDAC cell lines into age-matched C57BL/6 mice. (E-G) Tumor growth curve of mice in individual groups is shown. Initial PDAC cells were implanted subcutaneously, followed by

immunotherapy. The mice given immunotherapy comprising the combination of anti-TNFR2 and anti-PD-L1 antibodies were rechallenged by implantation of a second round of syngeneic PDAC cells from either the same cell line as the first implantation or a different cell line. (H) Representative images showing tumors harvested from mice bearing rechallenged-KPC after initial KPC implantation with combination therapy.
