# Supplementary material for: Combination cancer immunotherapy targeting TNFR2 and PD-1/PD-L1 signaling reduces immunosuppressive effects in the microenvironment of pancreatic tumors
Source: J Immunother Cancer. 2022 Mar 8;10(3):e003982. doi: 10.1136/jitc-2021-003982 (PMC8906048; doi:10.1136/jitc-2021-003982)
Supplement: online supplemental file 2 [file jitc-2021-003982supp002.pdf]

A

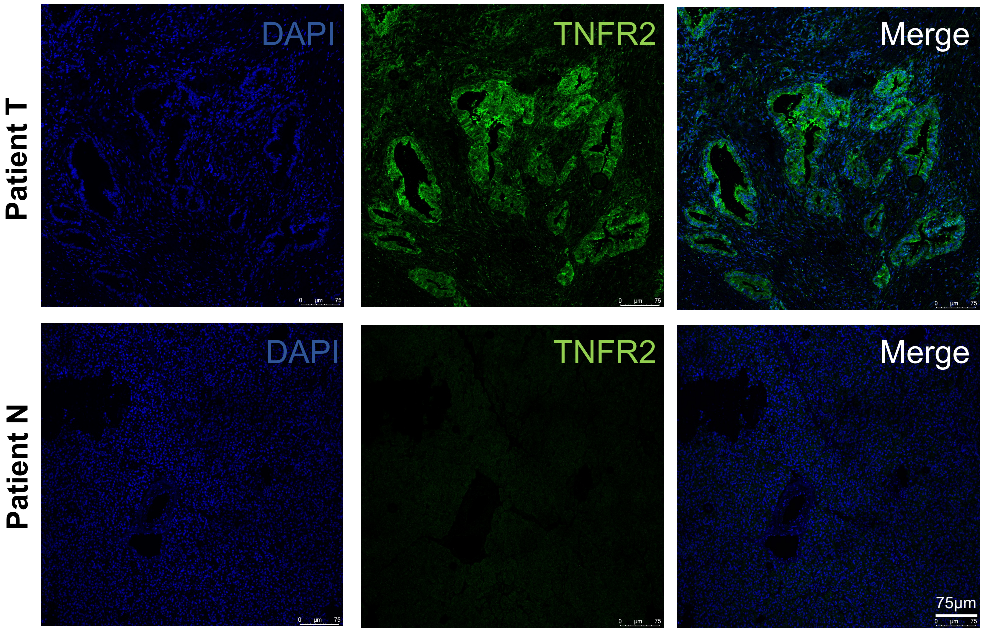

B

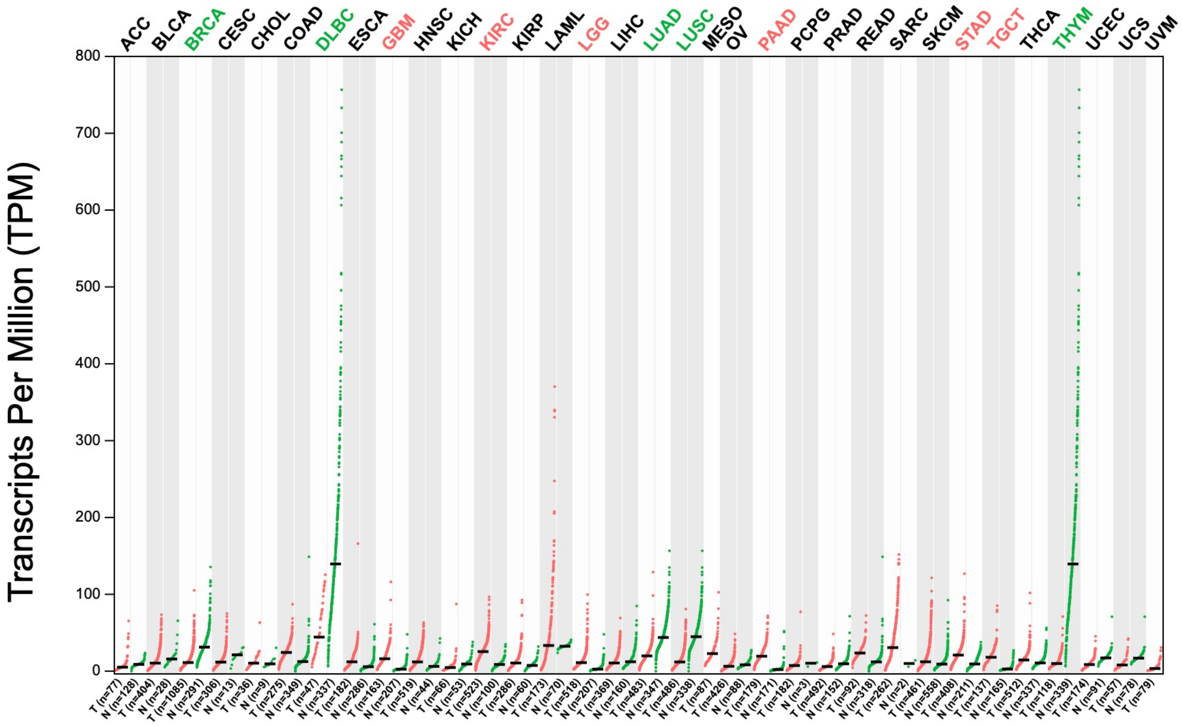

Supplementary Fig.1

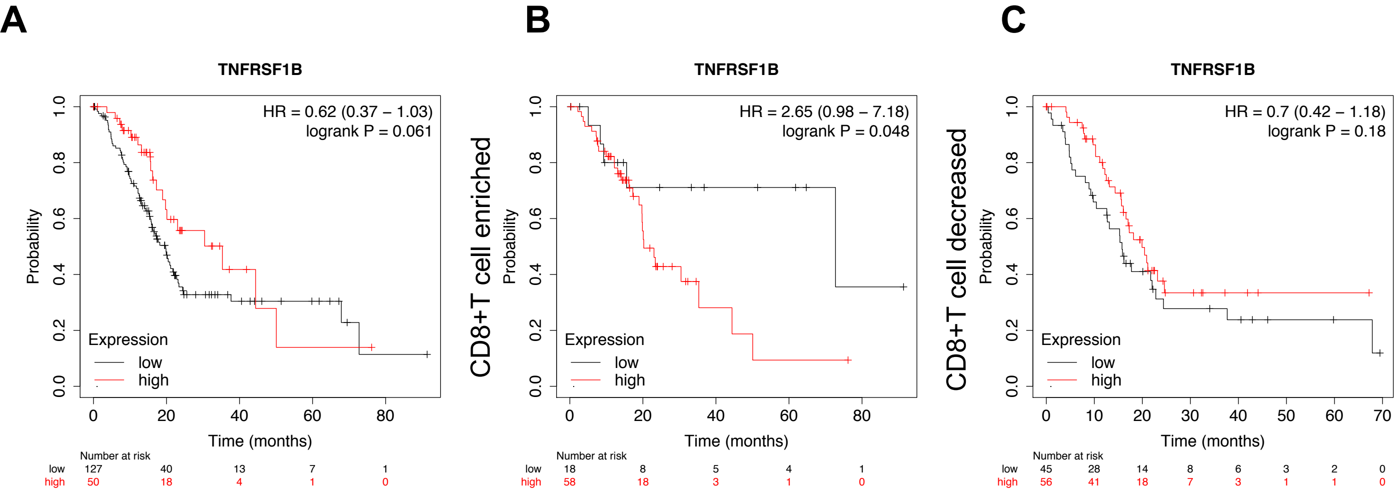

Supplementary Fig.2

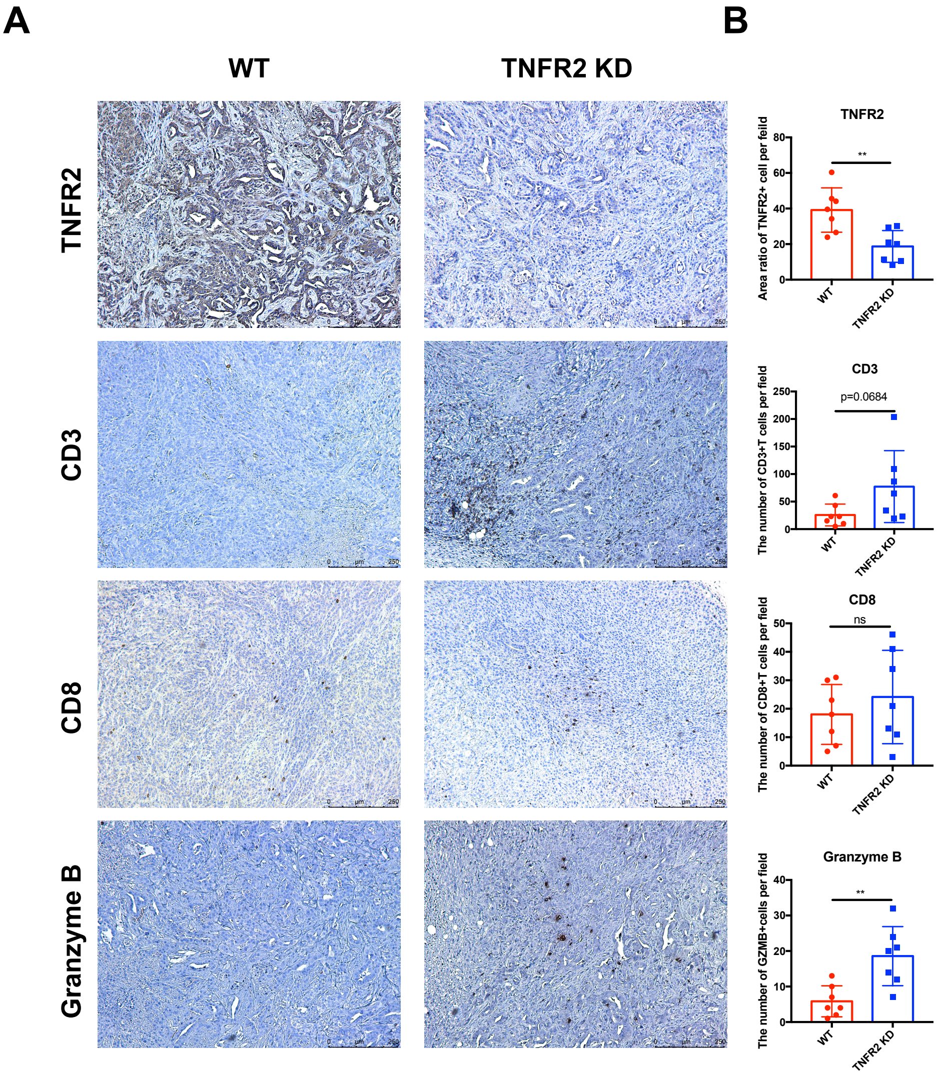

Supplementary Fig.3

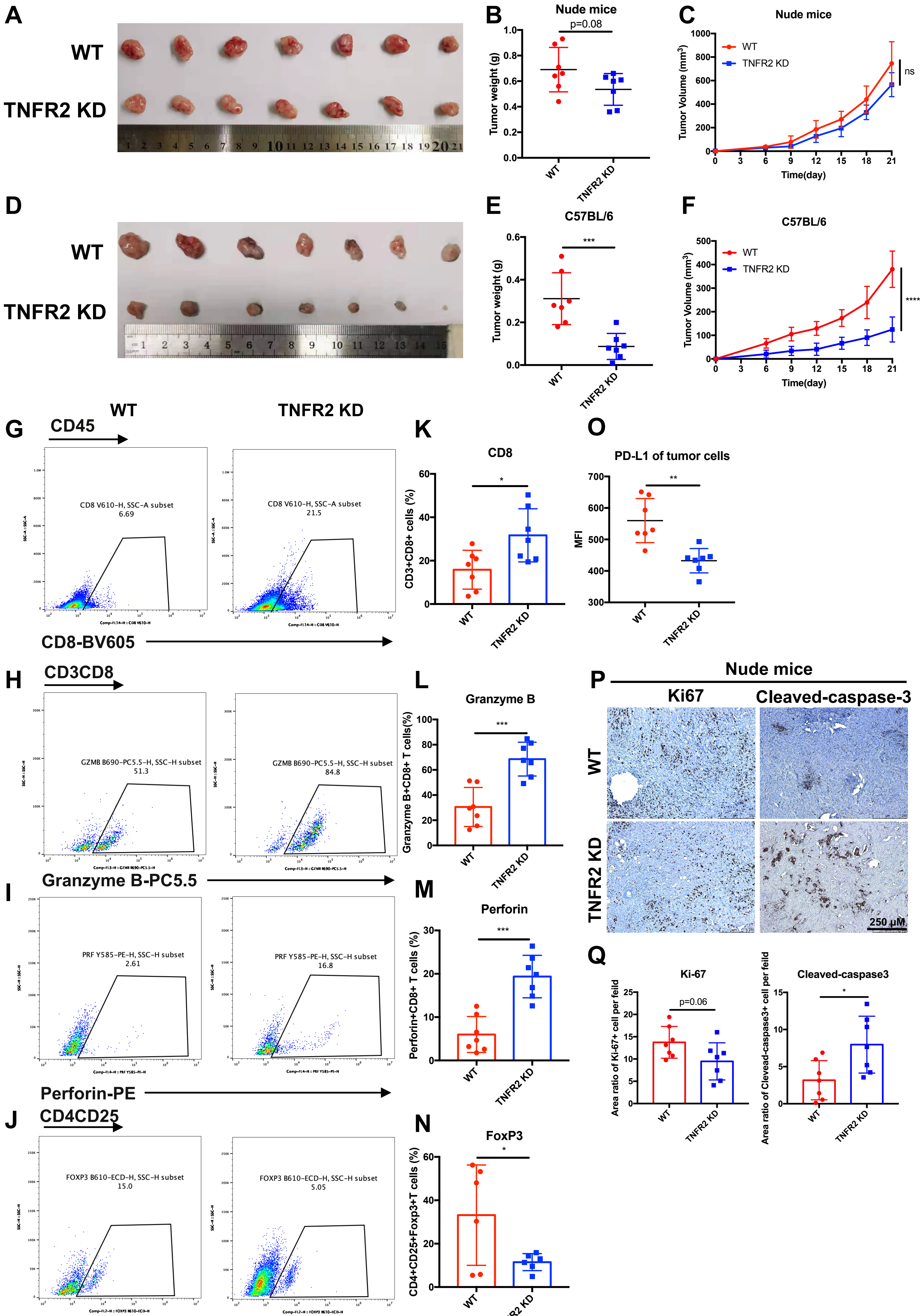

Supplementary Fig.4

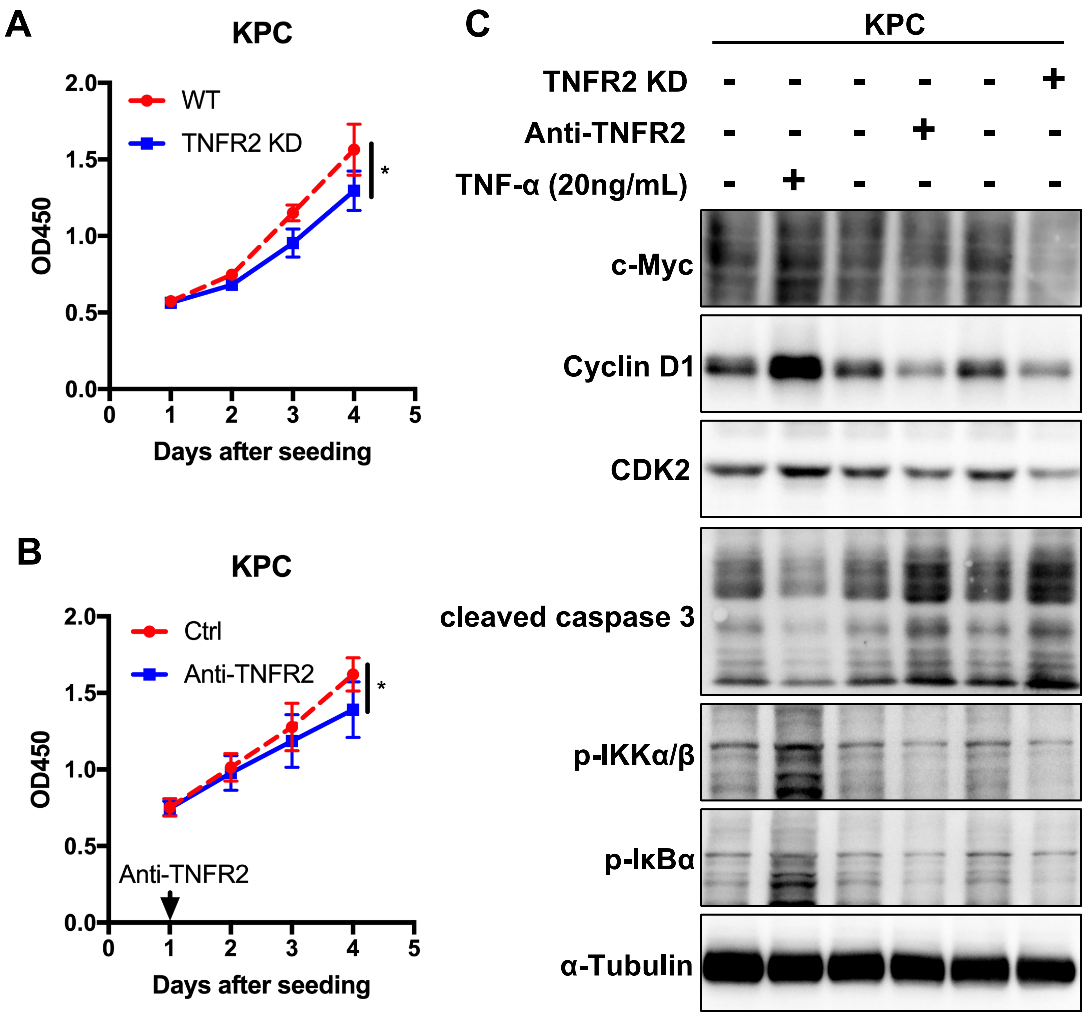

Supplementary Fig.5

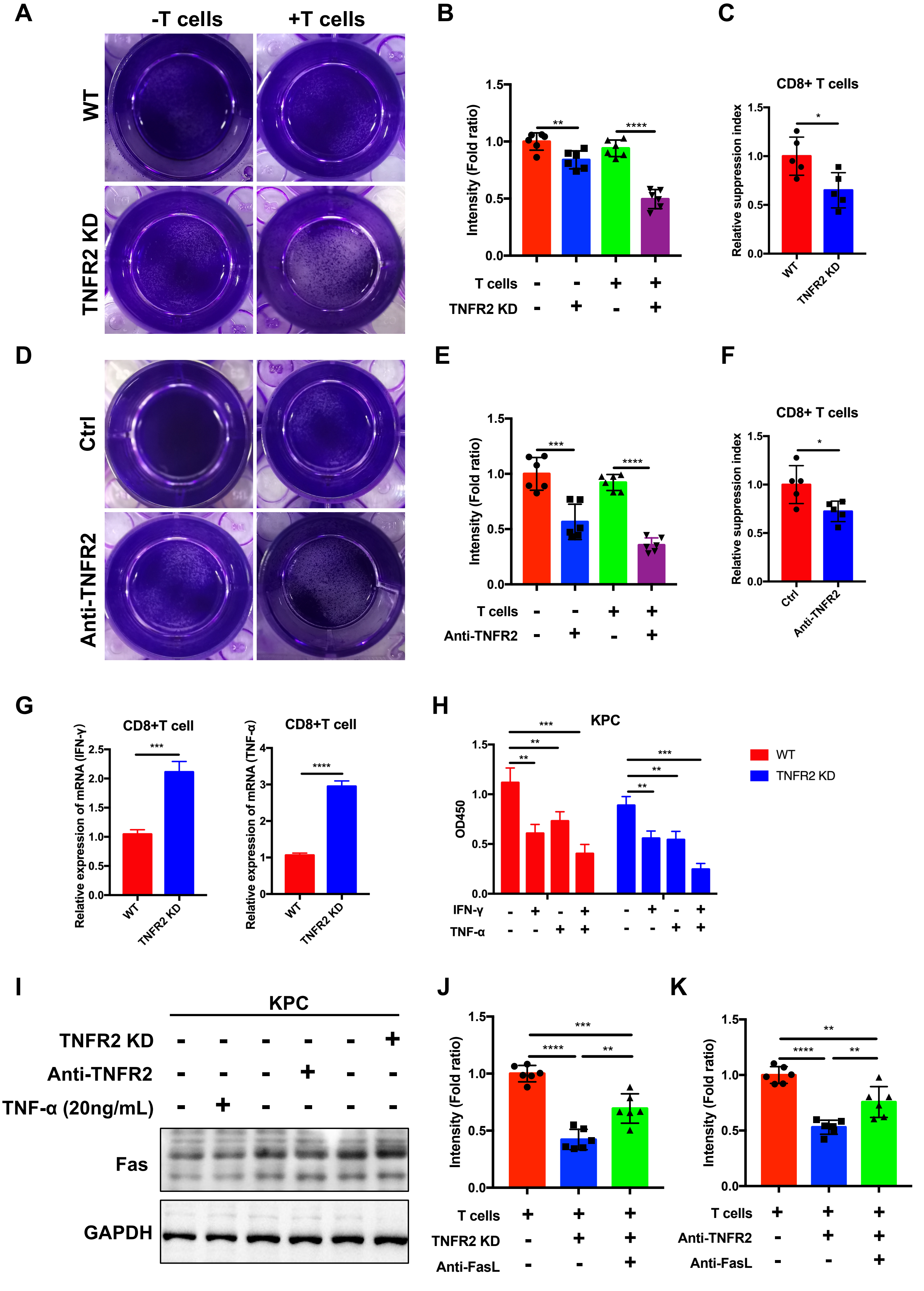

Supplementary Fig.6

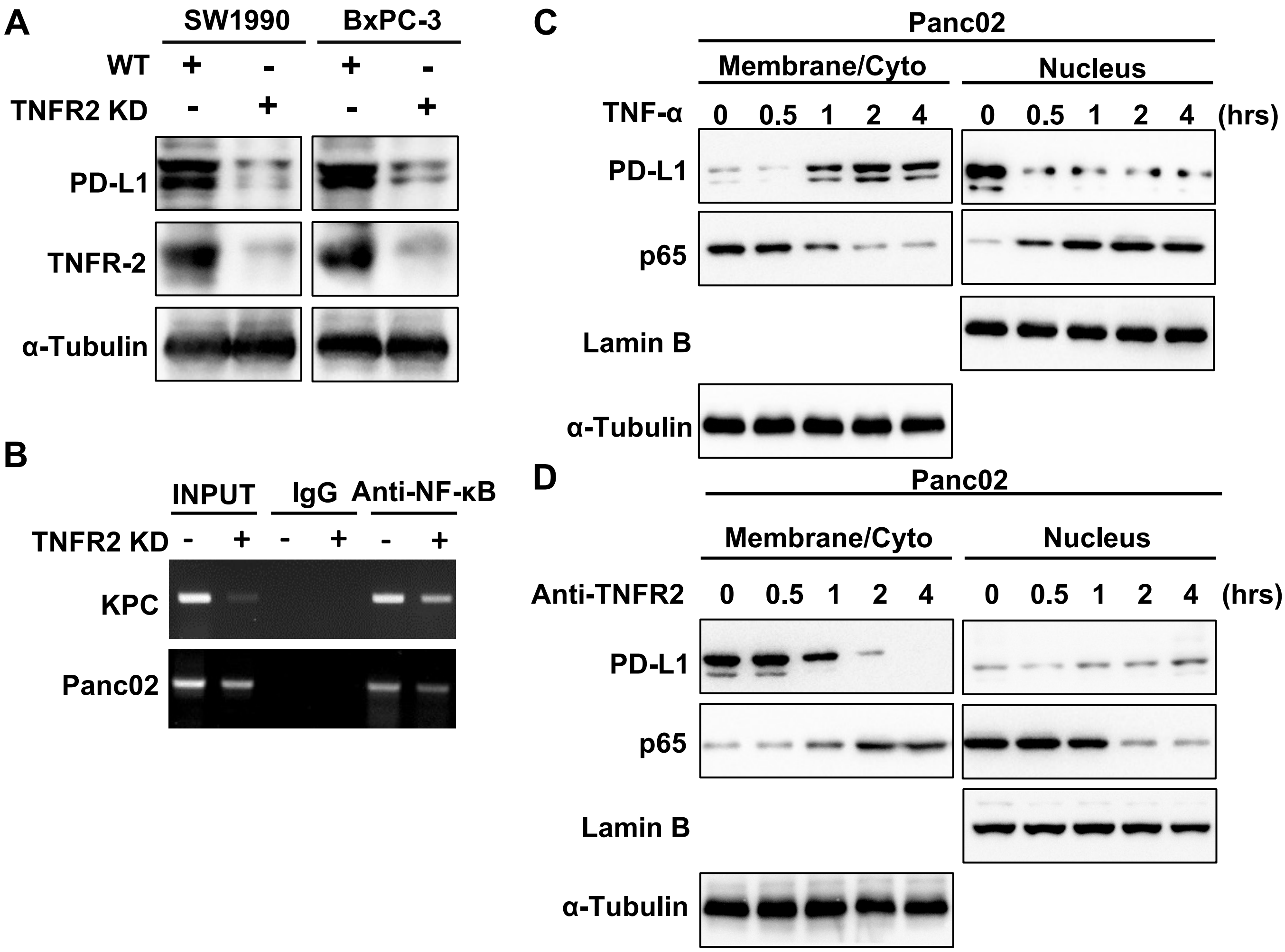

Supplementary Fig.7

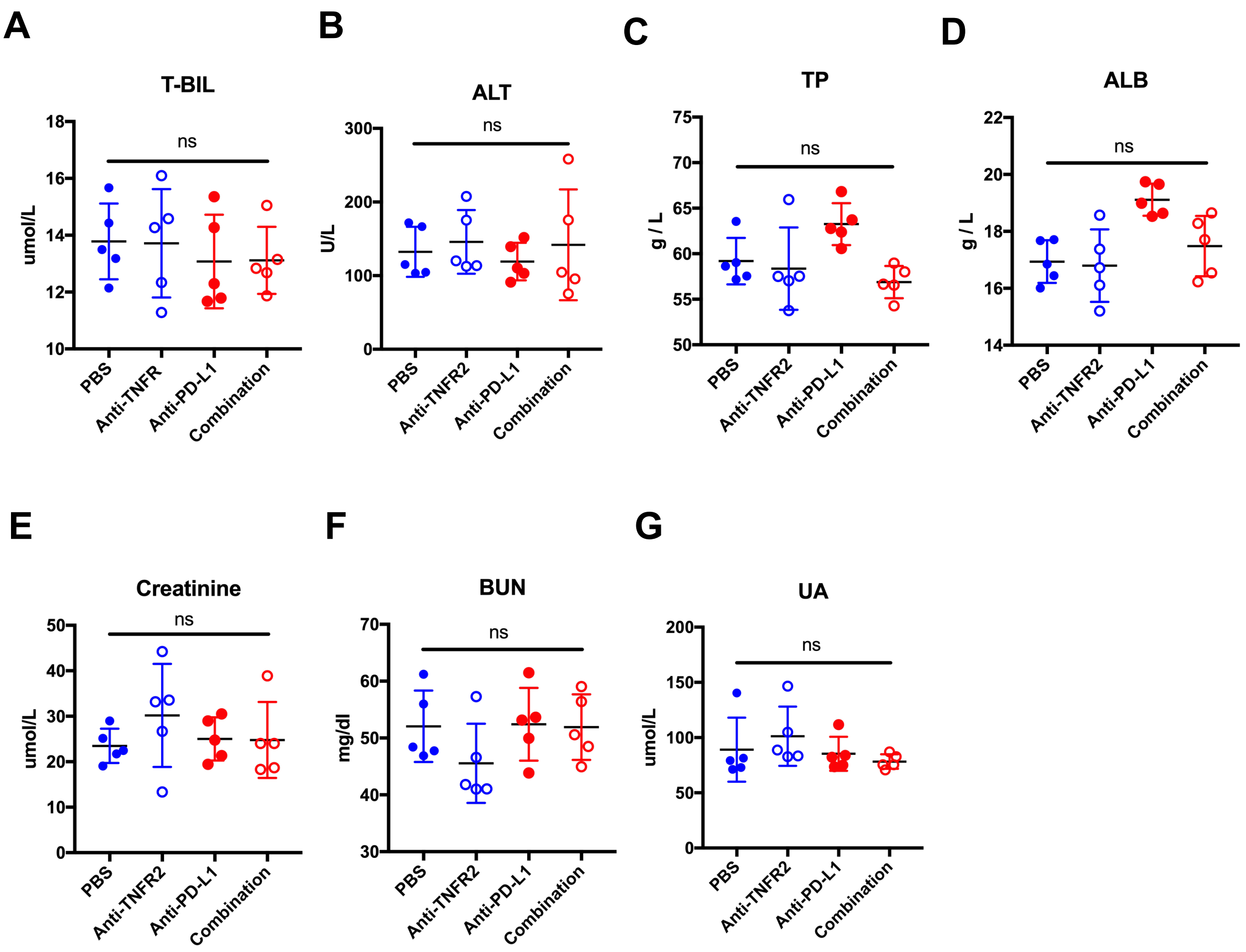

Supplementary Fig.8

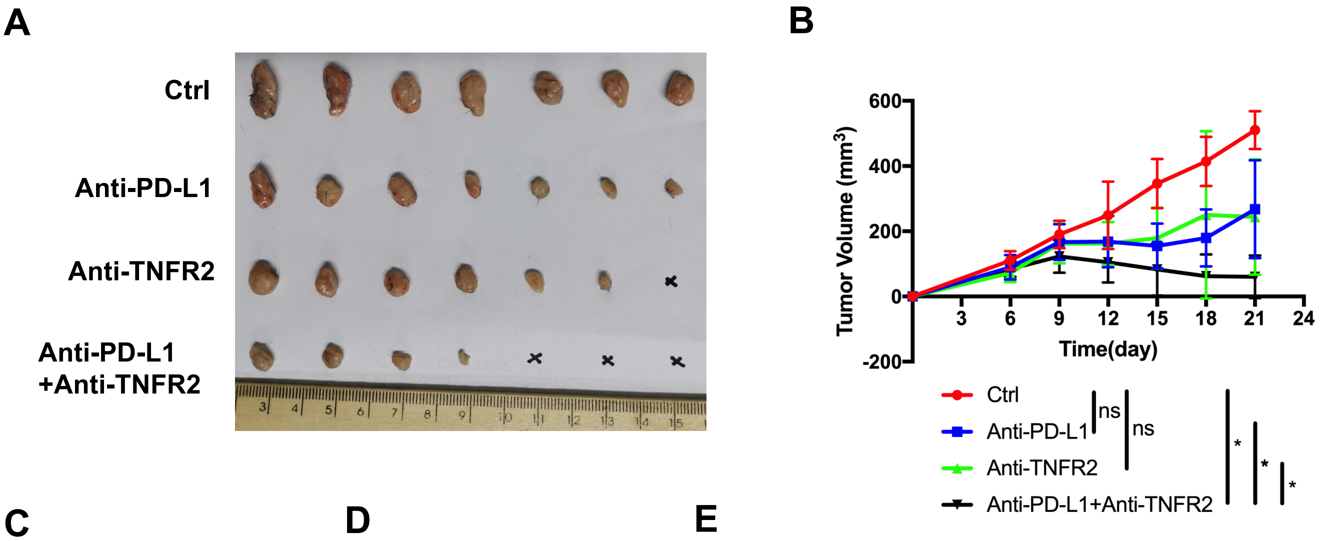

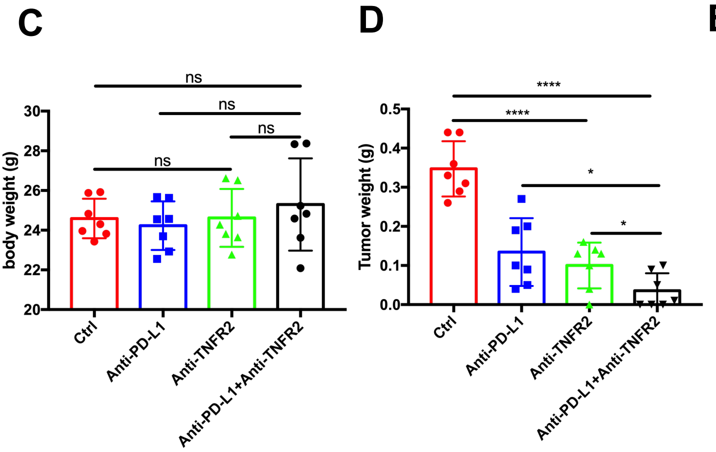

E

	Ctrl	Anti-PD-L1	Anti-TNFR2	Anti-PD-L1+Anti-TNFR2
Responders	0	1	3	7
Non-responders	21	20	18	14
Response rate (%)	**0.00**	**5.00**	**14.29**	**33.33**
Fisher's exact test (vs Ctrl)	N/A	> 0.999	0.232	0.009
Fisher's exact test (vs Anti-PD-L1		N/A	0.606	0.040

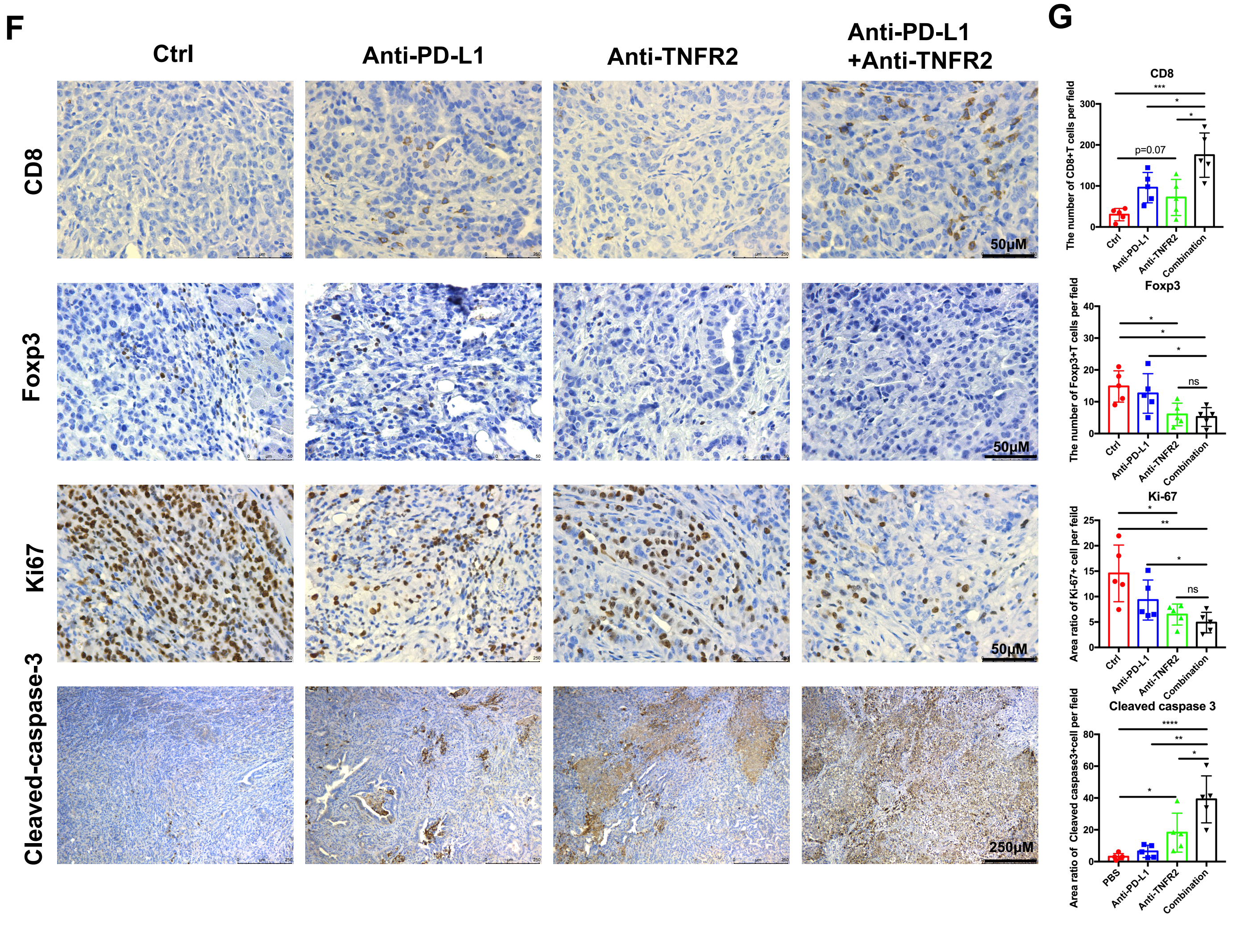

Supplementary Fig.9

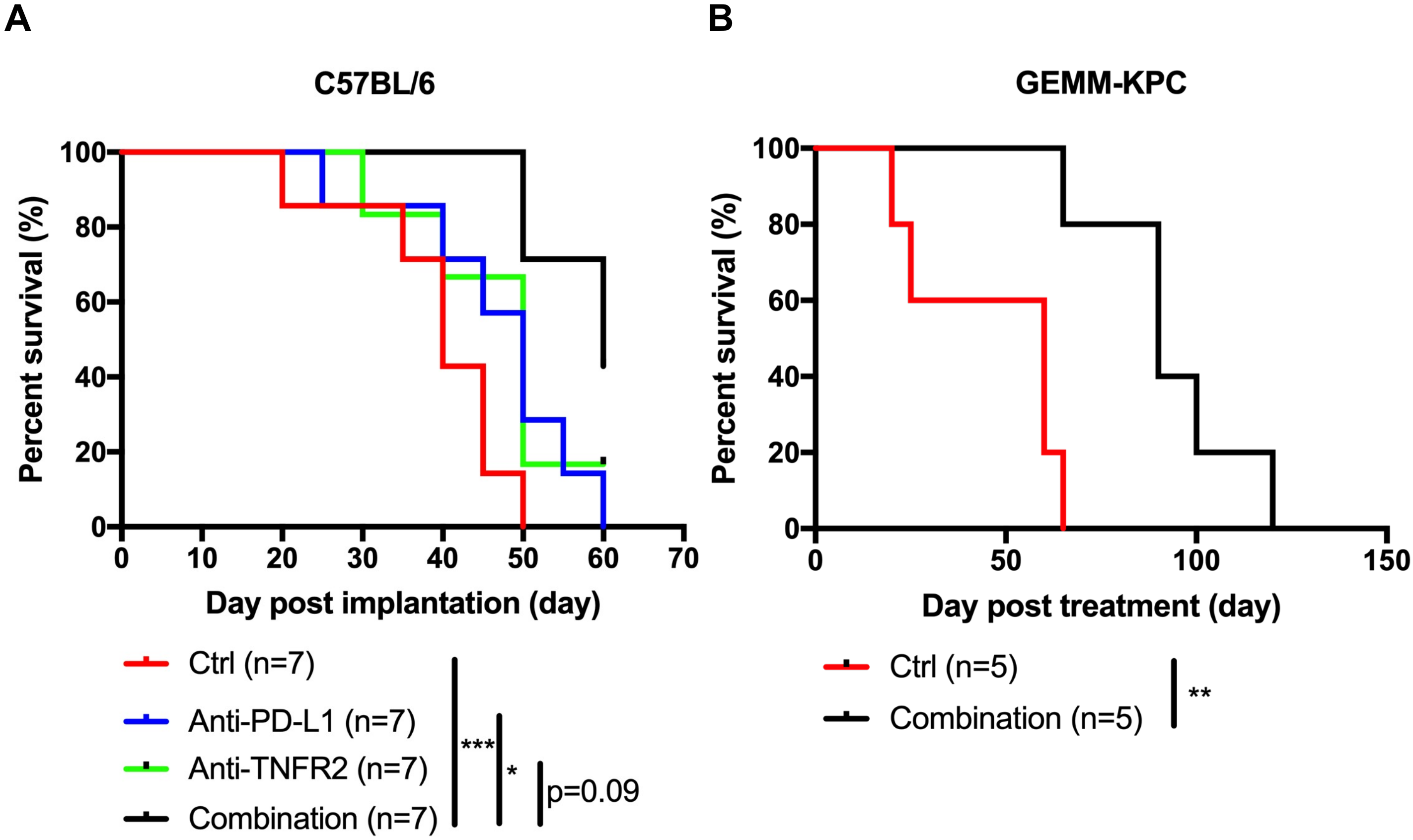

Supplementary Fig.10

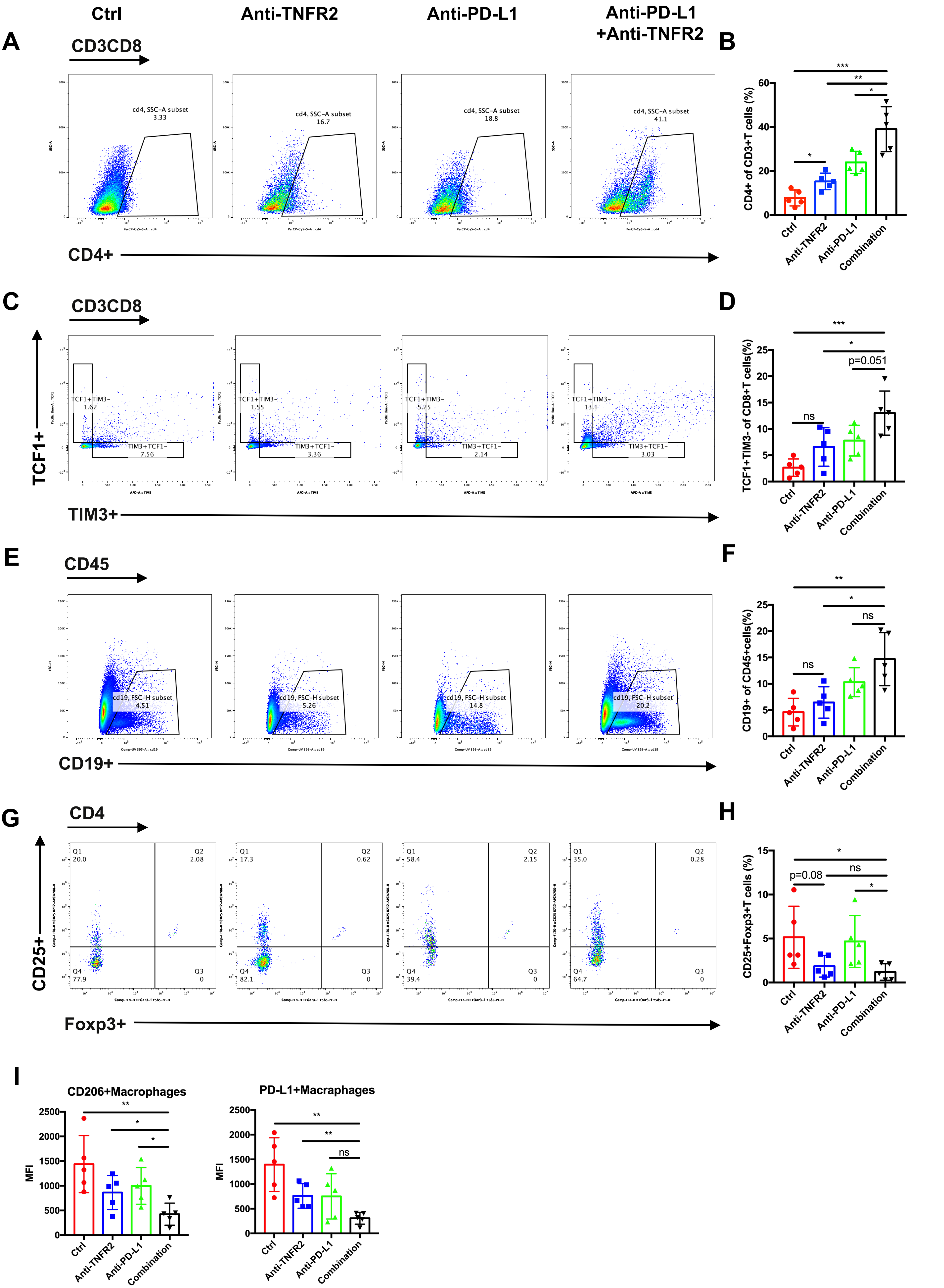

Supplementary Fig.11

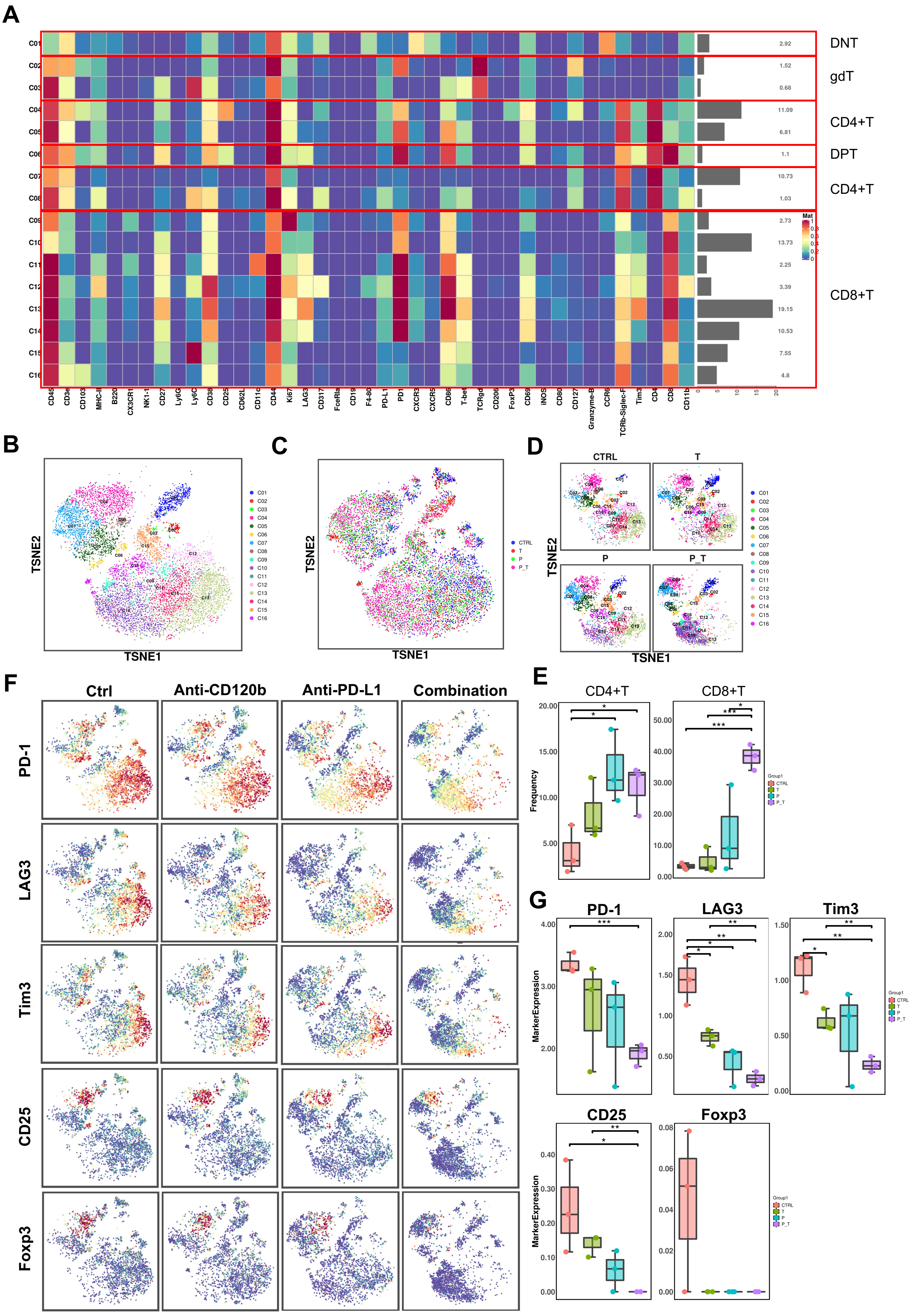

Supplementary Fig.12

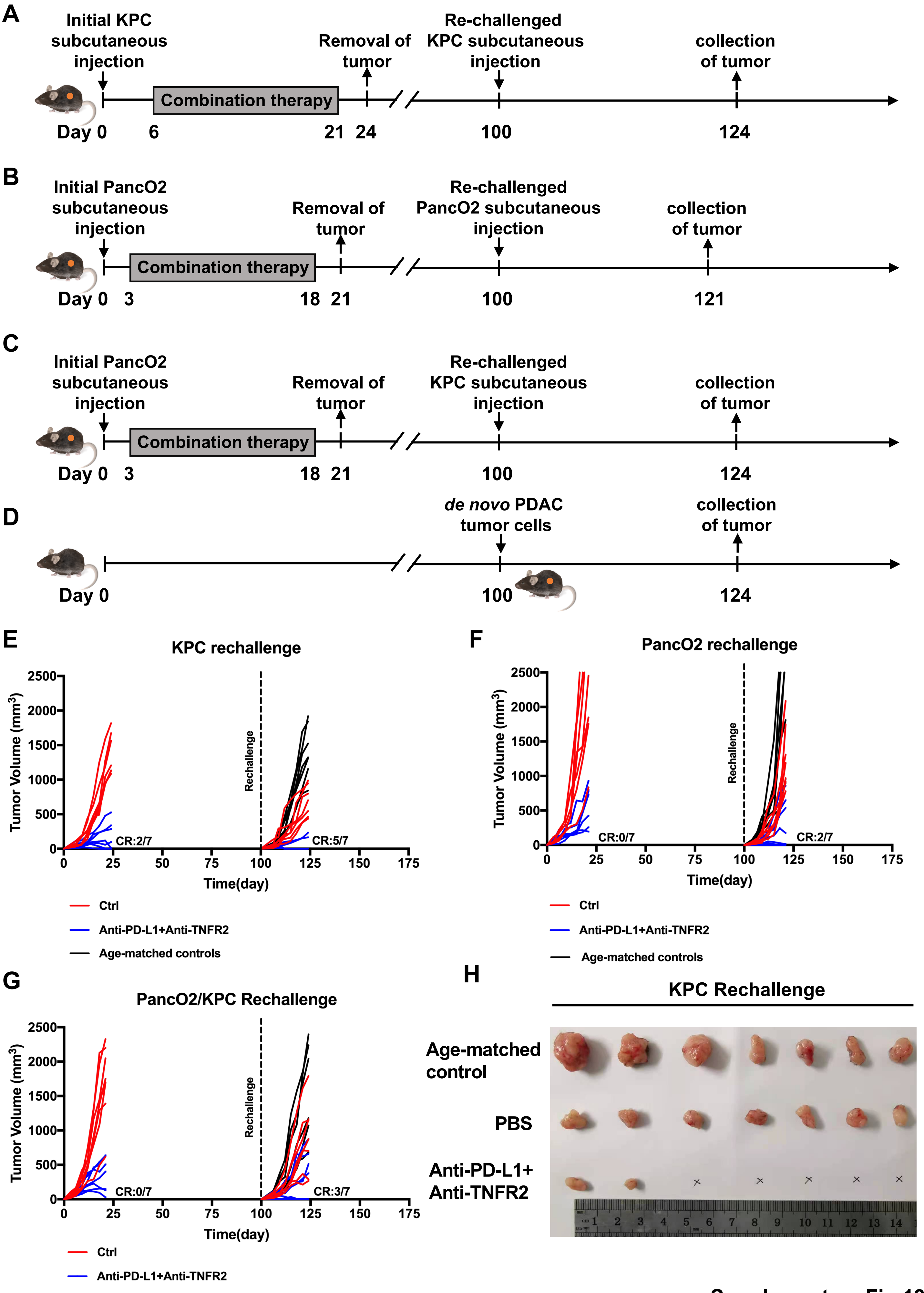

Supplementary Fig.13

Graphical Abstracts

Combination cancer immunotherapy targeting TNFR2 and PD-1/PD-L1 signaling reduces immunosuppressive effects in the microenvironment of pancreatic tumors

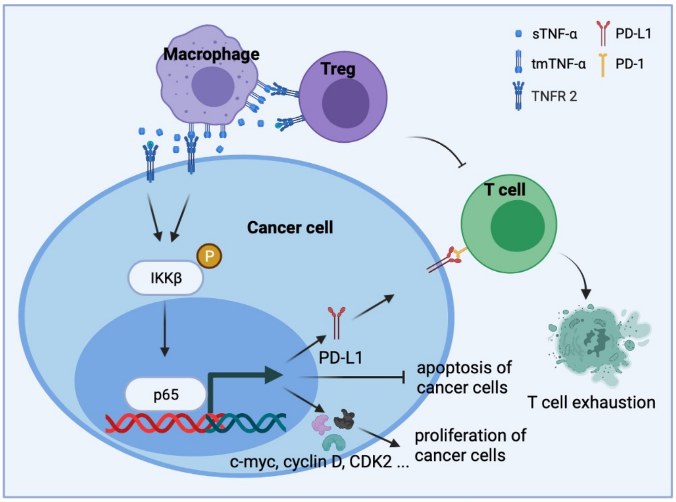

Authors

Xiaozhen Zhang, Mengyi Lao, Jian Xu, Yi Duan, Hanshen Yang, Muchun Li, Honggang Ying, Lihong He,Kang Sun, Chengxiang Guo, Wen Chen, Haitao Jiang, Xiaoyu Zhang, Xueli Bai, Tingbo Liang

Correspondence

liangtingbo@zju.edu.cn (T-B.L.);

shirleybai@zju.edu.cn (X-L.B.)

In Brief

TNFR2 regulate the expression of PD-L1 expression at the transcription level via the p65 NF-κB pathway. Anti-TNFR2 and anti-PD-L1 combination therapy eradicated tumors by inhibiting their growth, relieving tumor immunosuppression, and generating robust memory recall.
