# Supplementary material for: Combination cancer immunotherapy targeting TNFR2 and PD-1/PD-L1 signaling reduces immunosuppressive effects in the microenvironment of pancreatic tumors
Source: J Immunother Cancer. 2022 Mar 8;10(3):e003982. doi: 10.1136/jitc-2021-003982 (PMC8906048; doi:10.1136/jitc-2021-003982)
Supplement: online supplemental file 3 [file jitc-2021-003982supp003.pdf]

Supplementary Table 1. Clinicopathological relevance of TNFR2 in patients with PDAC

Variable	TMA TNFR2			Serum TNFR2		
	Low (H-score < 40)	High (H-score ≥ 40)	*P* value	Low (< 357.6 pg/mL)	High (≥ 357.6 pg/mL)	*P* value
Gender			**0.013**			0.140
male, n (%)	17 (38.6)	21 (67.7)		13 (48.1)	24 (66.7)	
female, n (%)	27 (61.4)	10 (32.3)		14 (51.9)	12 (33.3)	
Age, years			0.197			0.440
> 60, n (%)	33 (75.0)	27 (87.1)		19 (86.4)	21 (77.8)	
≤ 60, n (%)	11 (25.0)	4 (12.9)		3 (13.6)	6 (22.2)	
BMI, kg/m^2			0.344			**0.000**
< 18.5, n (%)	5 (13.2)	4 (13.3)		1 (4.8)	61 (22.2)	
18.5–23.9, n (%)	18 (47.4)	19 (63.3)		19 (90.4)	6 (22.2)	
> 23.9, n (%)	15 (39.4)	7 (23.3)		1 (4.8)	15 (55.6)	
TNM stage			**0.000**			**0.048**
I, n (%)	25 (58.1)	4 (12.9)		11 (17.3)	5 (29.7)	
II, n (%)	14 (32.6)	22 (71.0)		9 (50.7)	15 (39.2)	
III-IV, n (%)	4 (9.3)	5 (16.1)		2 (32.0)	7 (31.1)	
Vascular invasion			0.561			0.482
YES, n (%)	24 (54.5)	19 (61.3)		12 (54.5)	12 (44.4)	
NO, n (%)	20 (45.5)	12 (38.7)		10 (45.5)	15 (55.6)	
Nerve invasion			0.573			0.966
YES, n (%)	30 (63.2)	23 (77.9)		5 (22.7)	6 (22.2)	
NO, n (%)	14 (36.8)	8 (22.1)		17 (77.3)	21 (77.8)	
Serum CA12-5, U/mL			0.902			0.738
≥ 35, n (%)	10 (23.8)	7 (22.6)		17 (77.3)	19 (73.1)	
< 35, n (%)	32 (76.2)	24 (77.4)		5 (22.7)	7 (26.9)	
Serum CA19-9, U/mL			0.726			0.348
≥ 37, n (%)	34 (77.3)	25 (80.6)		20 (90.9)	22 (81.5)	
< 37, n (%)	10 (22.7)	6 (19.4)		2 (9.1)	5 (18.5)	
Serum CEA, U/mL			0.263			0.567
≥ 5, n (%)	19 (45.2)	10 (32.3)		14 (63.6)	15 (55.6)	
< 5, n (%)	23 (54.8)	21 (67.7)		8 (36.4)	12 (44.4)	
Tumor differentiation			0.757			0.289
well, n (%)	2 (4.8)	2 (6.7)		1 (4.8)	0 (0.0)	
moderate, n (%)	24 (57.1)	19 (63.3)		11 (52.3)	19 (70.4)	
poor, n (%)	16 (38.1)	9 (30.0)		9 (42.9)	8 (29.6)	
Recurrence			0.698			0.395
YES, n (%)	18 (54.5)	12 (60.0)		10 (55.6)	7 (41.2)	
NO, n (%)	15 (45.5)	8 (40.0)		8 (44.4)	10 (58.8)	
