# Supplementary material for: Combination cancer immunotherapy targeting TNFR2 and PD-1/PD-L1 signaling reduces immunosuppressive effects in the microenvironment of pancreatic tumors
Source: J Immunother Cancer. 2022 Mar 8;10(3):e003982. doi: 10.1136/jitc-2021-003982 (PMC8906048; doi:10.1136/jitc-2021-003982)
Supplement: online supplemental file 4 [file jitc-2021-003982supp004.pdf]

Supplementary Table 2: Table of key resources

Reagent or resource	Source	Identifier
**Antibodies**		
Anti-TNFR2 antibody	Abcam	ab109322
Anti-PD-L1 antibody	Thermo Fisher Scientific	24171-1-AP
Anti-PD-L1 antibody	Cell Signaling Technology	13684
Anti-PD-L1 antibody	Abcam	ab213480
Anti-PD-L1 antibody	Abcam	ab205921
Anti-PD-L1 antibody	Proteintech Group	66248-1-Ig
Anti-CD8a antibody	Cell Signaling Technology	98941
Anti-NF-κB p65 antibody	Cell Signaling Technology	6956T
Anti-Phospho-IKKα/β	Cell Signaling Technology	2697T
Anti-Phospho-IκBα	Cell Signaling Technology	2859T
Anti-Ki-67 antibody	Cell Signaling Technology	10020
Anti-Cleaved Caspase-3	Cell Signaling Technology	9664
Anti-Foxp3 antibody	Thermo Fisher Scientific	14-5773-82
GAPDH antibody	Beyotime	AF5009
Anti-Lamin B1 antibody	Abcam	ab229025
Anti-beta Actin antibody	Abcam	ab8226
Anti-Cyclin D1 antibody	Abcam	ab134175
Anti-c-Myc antibody	Abcam	ab32072
Anti-Cdk2 antibody	Abcam	ab32147
Anti-Fas antibody	Abcam	ab133619
mouse IgG Isotype Control	Santa Cruz	sc-2025
rabbit IgG Isotype Control	Cell Signaling Technology	8726S
HRP Goat Anti-Rabbit IgG	Beyotime	A0208
HRP Goat Anti-mouse IgG	Beyotime	A0216
Goat Anti-Rabbit IgG antibody	GeneTex	GTX77061
Goat Anti-Mouse IgG antibody	GeneTex	GTX26708
Anti-rabbit IgG (H+L), F(ab')2 Fragment (Alexa Fluor® 488 Conjugate)	Cell Signaling Technology	4412
Anti-mouse IgG (H+L), F(ab')2 Fragment (Alexa Fluor® 555 Conjugate)	Cell Signaling Technology	4409
PE Rat anti-Mouse Foxp3	BD biosciences	560408
BV421 Mouse Anti-TCF-7/TCF-1	BD biosciences	566692
BUV395 Rat Anti-Mouse CD19	BD biosciences	563557
PE-CF594 Rat Anti-CD11b	BD biosciences	562287
FITC anti-mouse Ki-67 Antibody	Biolegend	652410
BV421 Hamster Anti-Mouse CD120b	Biolegend	564088
FITC anti-mouse CD3 Antibody	Biolegend	100203
APC/Cy7 anti-mouse CD3 antibody	Biolegend	100329
Brilliant Violet 785 anti-mouse CD45 antibody	Biolegend	103111

PE/Cy7 anti-mouse CD8a antibody	Biolegend	100722
APC/Cyanine7 anti-mouse CD4 Antibody	Biolegend	100414
Alexa Fluor® 700 anti-mouse CD25 Antibody	Biolegend	102024
PE anti-mouse NK-1.1 Antibody	Biolegend	108708
FITC anti-mouse IFN-γ antibody	Biolegend	505806
APC anti-mouse CD49b (pan-NK cells) Antibody	Biolegend	108909
PerCP/Cyanine5.5 anti-human/mouse Granzyme B Recombinant Antibody	Biolegend	372212
PE anti-mouse Perforin antibody	Biolegend	154306
APC anti-mouse TNF-α antibody	Biolegend	506308
PE anti-mouse CD274 antibody	Biolegend	124308
FITC anti-mouse CD326 (Ep-CAM) antibody	Biolegend	118208
APC-anti-mouse CD366 (Tim-3) antibody	Biolegend	119706
PE/Cyanine7 anti-mouse F4/80 Antibody	Biolegend	123114
APC anti-mouse CD206 (MMR) Antibody	Biolegend	141708
Brilliant Violet 421 anti-human CD274 antibody	Biolegend	329714
PE Rat IgG2b, κ Isotype Ctrl Antibody	Biolegend	400607
Brilliant Violet 421 Mouse IgG2b, κ Isotype Ctrl antibody	Biolegend	400342
Trustain fcX anti-mouse CD16/CD32	Biolegend	101320
Human TruStain FcX	Biolegend	422302
Ultra-LEAF™ Purified anti-Asialo-GM1 Antibody	Biolegend	146002
Ultra-LEAF™ Purified anti-mouse CD120a (TNF R Type I/p55) Antibody	Biolegend	112906
Ultra-LEAF™ Purified anti-mouse CD120b (TNF R Type II/p75) Antibody	Biolegend	113305
CD3 Monoclonal Antibody (17A2), Functional Grade	eBioscience	16-0032-82
CD28 Monoclonal Antibody (37.51), Functional Grade	eBioscience	16-0281-82
InVivoMAb anti-mouse PD-L1	BioXcell	BE0101
InVivoMAb rat IgG2b isotype control	BioXcell	BE0090
InVivoMAb anti-mouse TNFR2	BioXcell	BE0247
InVivoMAb anti-mouse CD8a	BioXcell	BP0061

InVivoMAb anti-mouse CD4	BioXcell	BE0003
InVivoMAb anti-mouse FasL	BioXcell	BE0319
**Biological samples**		
Human PDAC tissue microarrays	The First Affiliated Hospital, School of Medicine, Zhejiang University	N/A
Paraffin sections from patients with PDAC	The First Affiliated Hospital, School of Medicine, Zhejiang University	N/A
Human PDAC serum samples	The First Affiliated Hospital, School of Medicine, Zhejiang University	N/A
KPC/KTC paraffin sections	This paper	N/A
**Chemicals, peptides, and recombinant proteins**		
BAY 11-7082	Selleck	S2913
JSH-23	Selleck	S7351
Recombinant Murine TNF-α	Peprotech	315-01A
Recombinant Murine IFN-γ	Peprotech	315-05
Recombinant Murine IL-2	Peprotech	212-12
Leukocyte Activation Cocktail	BD biosciences	550583
Percoll solution	GE healthcare	17-0891-01
Dynabeads Mouse T-Activator CD3/CD28	Thermo Fisher Scientific	11452D
Collagenase IV	Thermo Fisher Scientific	17104019
Dispase	Gibco	17105041
DNase	Sigma-Aldrich	D5025
Calcium chloride solution	Sigma-Aldrich	21115
Puromycin	Invivogen	ant-pr-1
Hoechst	Solarbio	C0021
Protease Inhibitor Cocktail	Bimake	B14001
Phosphatase Inhibitor Cocktail	Bimake	B15001
**Critical commercial assays**		
Fixation/Permeabilization Solution Kit	BD biosciences	555028
Fixable Viability Stain 780	BD biosciences	565388
DAB Chromogen Kit	Biocare	BDB2004
CFSE Cell Division Tracker Kit	Biolegend	423801
LIVE/DEAD™ Fixable Violet Dead Cell Stain Kit, for 405 nm excitation	Thermo Fisher Scientific	L34963
Nuclear and Cytoplasmic Extraction Reagent	Thermo Fisher Scientific	78833
eBioscience™ Foxp3 / Transcription Factor Staining Buffer Set	Invitrogen	00-5523-00

Human sTNF RII/TNFRSF1B Quantikine ELISA Kit	R&D	DRT200
CD8a+ T Cell Isolation Kit, mouse	miltenyibiotec	130-104-075
**Deposited data**		
CyTOF analysis	This paper	N/A
**Experimental models: cell lines**		
KPC	KrasG12D;Trp53R172H;Pdx1-Cre (KPC) mice	N/A
Panc02	ATCC	CRL-2553
SW1990	ATCC	CRL-2172
BxPC-3	ATCC	CRL-1687
**Experimental models: organisms/strains**		
C57BL/6	Model animal research center of Nanjing University	N/A
Nude mice	Model animal research center of Nanjing University	N/A
GEMM-KPC	Laboratory of Prof. Raghu Kalluri (MD Anderson Cancer Center, Houston, TX, USA)	N/A
**Recombinant DNA**		
Human TNFR2 knockdown lentivirus	Shanghai OBiO Technology	N/A
Mouse *Tnfr2* knockdown lentivirus	Shanghai OBiO Technology	N/A
**Software and algorithms**		
GraphPad Prism 7.0	GraphPad Software, Inc	N/A
FlowJo 10.0	BD Life Sciences	N/A
Image J 1.8.0	National Institutes of Health	N/A
