# Supplementary material for: Combination cancer immunotherapy targeting TNFR2 and PD-1/PD-L1 signaling reduces immunosuppressive effects in the microenvironment of pancreatic tumors
Source: J Immunother Cancer. 2022 Mar 8;10(3):e003982. doi: 10.1136/jitc-2021-003982 (PMC8906048; doi:10.1136/jitc-2021-003982)
Supplement: online supplemental file 5 [file jitc-2021-003982supp005.pdf]

Supplementary Table 3: List of Antibodies in CyTOF

List	Label	marker	clone	Company
1	89Y	CD45	30-F11	Biolegend
2	115ln	CD3e	145-2C11	Biolegend
3	141Pr	CD103	2E7	Biolegend
4	142Nd	MHC II	M5/114.15.2	Biolegend
5	143Nd	CD45R_B220	RA3-6B2	Biolegend
6	144Nd	CX3CR1	SA011F11	Biolegend
7	145Nd	CD161c_NK1.1	PK136	Biolegend
8	146Nd	CD27	LG.3A10	Biolegend
9	147Sm	Ly6G	1A8	Biolegend
10	148Nd	Ly6C	HK1.4	Biolegend
11	149Sm	CD38	90	Biolegend
12	150Nd	CD25_IL-2R	3C7	Biolegend
13	151Eu	CD62L	MEL14	Biolegend
14	152Sm	CD11c	N418	Biolegend
15	153Eu	CD44	IM7	Biolegend
16	154Sm	Ki67	SolA15	eBioscience
17	155Gd	CD223_LAG3	C9B7W	Biolegend
18	156Gd	CD317_BST2	44E9R	RD
19	157Gd	FceRIα	MAR-1	Biolegend
20	158Gd	CD19	6D5	Biolegend
21	159Tb	F4_80	C1:A3-1	BioRAD
22	160Gd	CD274_PD_L1	10F.9G2	Biolegend
23	161Dy	CD279_PD1	29F.1A12	Biolegend
24	162Dy	CD183_CXCR3	CXCR3-173	Biolegend
25	163Dy	CD185_CXCR5	L138D7	Biolegend
26	164Dy	CD86	GL-1	Biolegend
27	165Ho	T_bet	4B10	Biolegend
28	166Er	TCRgd	GL3	Biolegend
29	167Er	CD206_MMR	C068C2	Biolegend
30	168Er	FoxP3	FJK-16s	eBioscience
31	169Tm	CD69	H1.2F3	Biolegend
32	170Er	iNOS_NOS2	CXNFT	eBioscience
33	171Yb	CD80	16-10A1	Biolegend
34	172Yb	CD127_IL7Ra	A7R34	Biolegend
35	173Yb	Granzyme B	GB11	Biolegend
36	174Yb	CD196_CCR6	29-2L17	Biolegend
37	175Lu	TCRb	H57-597	Biolegend
38	175Lu	Siglec F	E50-2440	BD
39	176Yb	CD366_Tim3	RMT 3-23	Biolegend
40	197Au	CD4	RM4-5	Biolegend
41	198pt	CD8	53-6.7	Biolegend
42	209Bi	CD11b	M1/70	PLT
